# The genome of the ant *Tetramorium bicarinatum* reveals a tandem organization of venom peptides genes allowing the prediction of their regulatory and evolutionary profiles

**DOI:** 10.1186/s12864-024-10012-y

**Published:** 2024-01-20

**Authors:** Axel Touchard, Valentine Barassé, Jean-Michel Malgouyre, Michel Treilhou, Christophe Klopp, Elsa Bonnafé

**Affiliations:** 1 BTSB-UR 7417, Université Fédérale de Toulouse, Institut National Universitaire Jean-François Champollion, Place de Verdun, 81000 Albi, France; 2 Department of Entomology, Cornell University, Ithaca, NY 14853 USA; 3 INRAE, BioinfOmics, Université Fédérale de Toulouse, GenoToul Bioinformatics Facility, Sigenae, 31326 Castanet-Tolosan, France

**Keywords:** *Tetramorium bicarinatum*, Toxins, Peptides, Venom, Ants, Chromosome-level genome, Evolution, Expression control

## Abstract

**Background:**

Venoms have evolved independently over a hundred times in the animal kingdom to deter predators and/or subdue prey. Venoms are cocktails of various secreted toxins, whose origin and diversification provide an appealing system for evolutionary researchers. Previous studies of the ant venom of *Tetramorium bicarinatum* revealed several Myrmicitoxin (MYRTX) peptides that gathered into seven precursor families suggesting different evolutionary origins. Analysis of the *T. bicarinatum* genome enabling further genomic approaches was necessary to understand the processes underlying the evolution of these myrmicitoxins.

**Results:**

Here, we sequenced the genome of *Tetramorium bicarinatum* and reported the organisation of 44 venom peptide genes (*vpg*)*.* Of the eleven chromosomes that make up the genome of *T. bicarinatum*, four carry the *vpg* which are organized in tandem repeats*.* This organisation together with the ML evolutionary analysis of *vpg* sequences, is consistent with evolution by local duplication of ancestral genes for each precursor family. The structure of the *vpg* into two or three exons is conserved after duplication events while the promoter regions are the least conserved parts of the *vpg* even for genes with highly identical sequences. This suggests that enhancer sequences were not involved in duplication events, but were recruited from surrounding regions. Expression level analysis revealed that most *vpg* are highly expressed in venom glands, although one gene or group of genes is much more highly expressed in each family. Finally, the examination of the genomic data revealed that several genes encoding transcription factors (TFs) are highly expressed in the venom glands. The search for binding sites (BS) of these TFs in the *vpg* promoters revealed hot spots of GATA sites in several *vpg* families.

**Conclusion:**

In this pioneering investigation on ant venom genes, we provide a high-quality assembly genome and the annotation of venom peptide genes that we think can fosters further genomic research to understand the evolutionary history of ant venom biochemistry.

**Supplementary Information:**

The online version contains supplementary material available at 10.1186/s12864-024-10012-y.

## Background

The evolution of venom toxins is a fascinating theme that has attracted the attention of many researchers, as it provides insights into the molecular mechanisms underlying adaptation and diversification in biological systems. Venom is an adaptive trait that has evolved independently over a hundred times in the animal kingdom. These secreted biological substances confer venomous organisms fitness advantages in defending against predators, subduing prey, competing with opponents and more [1]. To these ends, venoms disrupt specific physiological systems through sophisticated mixtures composed of a variety of peptides and proteins, that independently and/or synergistically target a wide range of pharmacological receptors ultimately causing pain, paralysis, or death [2]. Beyond the intrinsic diversity of toxins, venom composition is even more complex as toxins are not present in equal proportions, further highlighting that evolution also affected the regulation of toxin-encoding genes to yield fine-tuned weapons [3].

So far, venom toxin evolution has mostly been investigated in snakes, tarantulas, scorpions, or cone snails while insights into the evolutionary dynamics of insect toxins are comparatively much sparser [4]. This knowledge gap is explained by the small size of insects and the difficulty of collecting large quantities of venom, which have long hindered the study of venom toxins. Another reason is that studies have focused on medically important animals. Insects rarely cause human death except in cases of anaphylactic shock and have therefore been left out of venom research. Importantly, next-generation sequencing has facilitated the study of venom composition from limited amount and opened new perspectives for the study of insect venoms. By combining venom proteomics and venom gland transcriptomics, numerous insect venom profiles (e.g., assassin bug) [5], limacodid caterpillar [6], assassin fly [7] have been published in recent years, broadening the sets of toxin peptide sequences. However, these proteo-transcriptomic studies rarely include genomic data yet being essential for understanding the evolution of toxins. For instance, the inclusion of genomic data has revealed that venom toxins undergo various genetic mechanisms in different insect lineages such asilid flies [8], parasitoid wasps [9], and recently bees [10].

Hymenoptera are the most speciose group of venomous organisms possessing a vast array of ecological, anatomical, and behavioural traits. Their venoms are involved not only in prey capture or self-defence as in most venomous animals, but also in colony defence against arthropod and vertebrate predators, control of microbial pathogens, communication (e.g., trail and alarm pheromones), venom detoxification and, in symbiotic ant-plant mutualisms in the elimination of plants that compete with their host myrmecophyte (i.e., plants that shelter ants in specific hollow structures) [11]. Parasitoid wasps also employ their venom to manipulate insect hosts in order to favour their offspring [12]. Consequently, hymenopteran venoms represent an interesting natural system to study the molecular evolution under different selective pressures. Hymenopteran venoms represent a new paradigm compared to iconic venomous animals (i.e., snakes, arachnids, scorpions, cone snails) because of their “simpler” composition. Except few examples [13], most proteo-transcriptomic studies on ants [11], bees [14], wasps [15], velvet ants [16] have indeed reported venoms composed of at most a few dozen toxins, largely dominated by small linear membrane-disrupting peptides. These peptides are often multifunctional, with antimicrobial, insecticidal, or algesic properties [17]. Robinson and colleagues noted that most of the venom peptide sequences described in aculeate Hymenoptera vary widely in their mature parts but shared similar pre-pro sequences suggesting a common origin and proposed to group these toxins into a single gene family called Aculeatoxins [13]. However, evolutionary analyses performed by Koladorov et al. on a dataset containing bee, wasp and ant venom peptides do not support this hypothesis, as ant venom peptides clustered differently from the others [10]. Thus, the evolution of Hymenoptera venoms appears to be much more complex than originally thought and warrants further investigations.

The present study aims to provide new insights into the evolution of ant venoms as no studies have been conducted on the genes encoding ant venom peptides. In this work, the genome of the ant *Tetramorium bicarinatum*, whose venom is one of the most extensively studied among ants, was sequenced and assembled into chromosomes. Previous studies have described the venom portrait of this species and provided a set of 37 venom peptides (matures and prepro-sequences) [18]. The precursors encoding these peptides were then classified into three major superfamilies tentatively named A, B and C based on prepro-sequence similarity and maturation profile. Briefly, genes belonging to group A are related to aculeatoxin superfamily of genes, group C genes encode for secapins while group B genes have been classified apart. Recently, another study showed a high turnover in the venom composition among the subfamily Myrmicinae with very heterogeneous venom peptide profiles of six species [19]. This extended dataset allowed a subdivision of the 37 venom peptide precursors of *T. bicarinatum* into seven families (A1, A2, A3, A4, B1, B2 and C1—for details see [18, 19]). These previous studies raised questions about the mechanisms underlying the recruitment and evolution of genes encoding these venom peptide families.

By leveraging previous venomic investigation of the ant *T. bicarinatum*, we described the venom peptides-encoding genes (i.e., chromosomal localization, structural arrangement, sequence conservation). In the light of this genomic data, we reanalyzed the expression level of genes encoding peptides in the venom of *T. bicarinatum* and searched for putative transcription factors controlling their expression in venom glands.

## Results

### Assembly quality

As shown in Additional_file_1 (Figure S0A), MaSurCa produced an assembly which total length of 276.3 Mb was close but above to the expected genome size given by genomescope2 of 250 Mb. This assembly had a N50 value of 3.4 Mb and a L50 value of 24. The BUSCO score (Additional_file_1, Figure S0B) of this assembly showed only 6 fragmented and 5 missing genes out of 1,658. After scaffolding, the genome size decreased to 258 Mb with a N50 value of 22.4 Mb and a L50 of 5. The corresponding BUSCO score showed only 7 fragmented and 5 missing genes out of 1,658. The decrease in duplicated genes, from 51 to 18, as well as the assembly size reduction of 18 Mb corresponded to the removal of retained haplotypic contigs in the contig assembly during scaffolding. The kmer content (Additional_file_1, Figure S0C) of the scaffolded assembly showed a very low fraction of kmer present in the reads and missing or in more than one copy in the assembly. The Hi-C map (Additional_file_1, Figure S0D) showed a very high link density within the eleven chromosomes when compared to link density between chromosomes. These metrics showed that both contig and chromosomal assemblies were of high quality.

### Localization and organization of venom peptide genes (vpg) loci

**Fig. 1:**
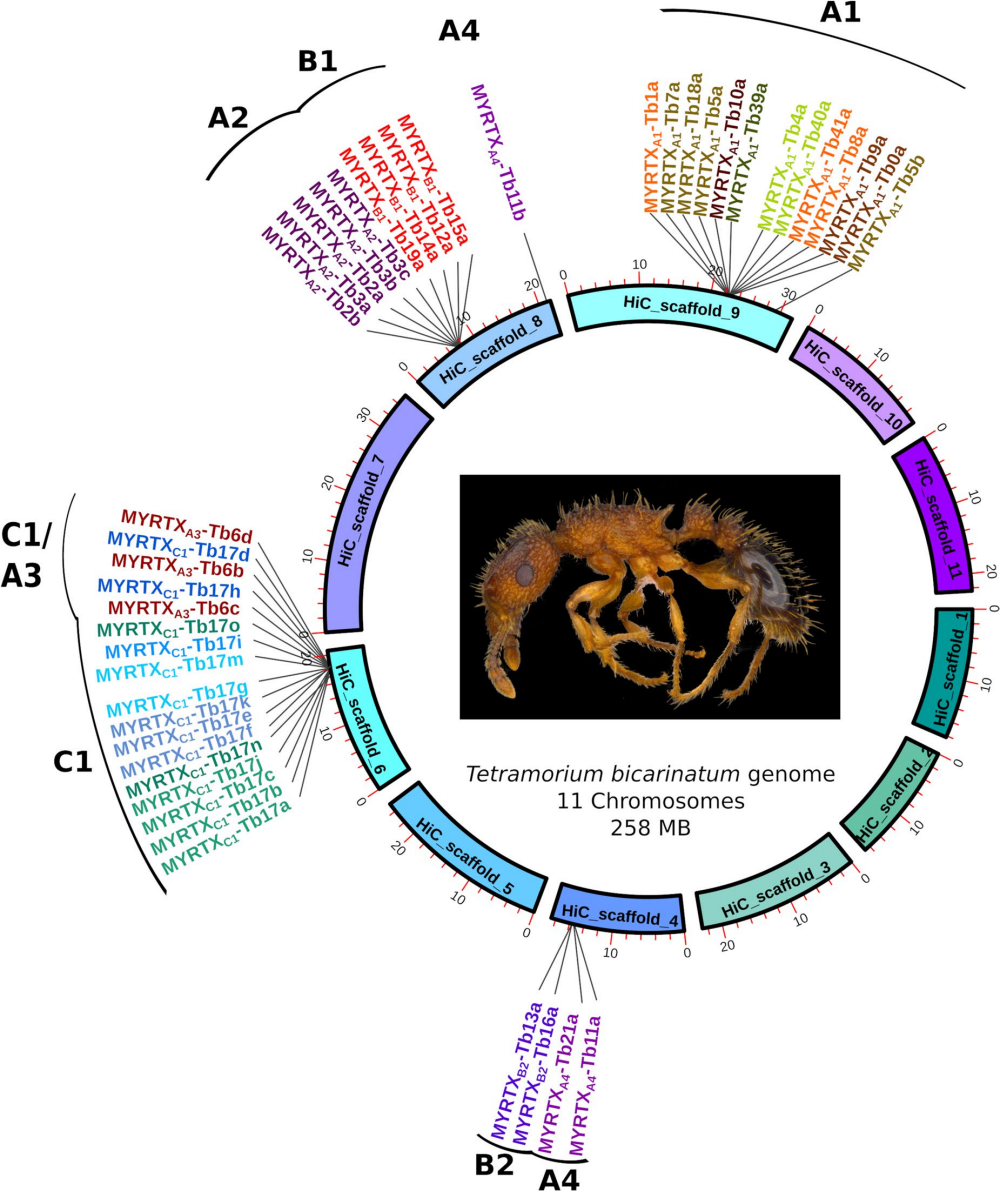
Location of venom peptide genes on the chromosomes of *T. bicarinatum* ant

**Fig. 2:**
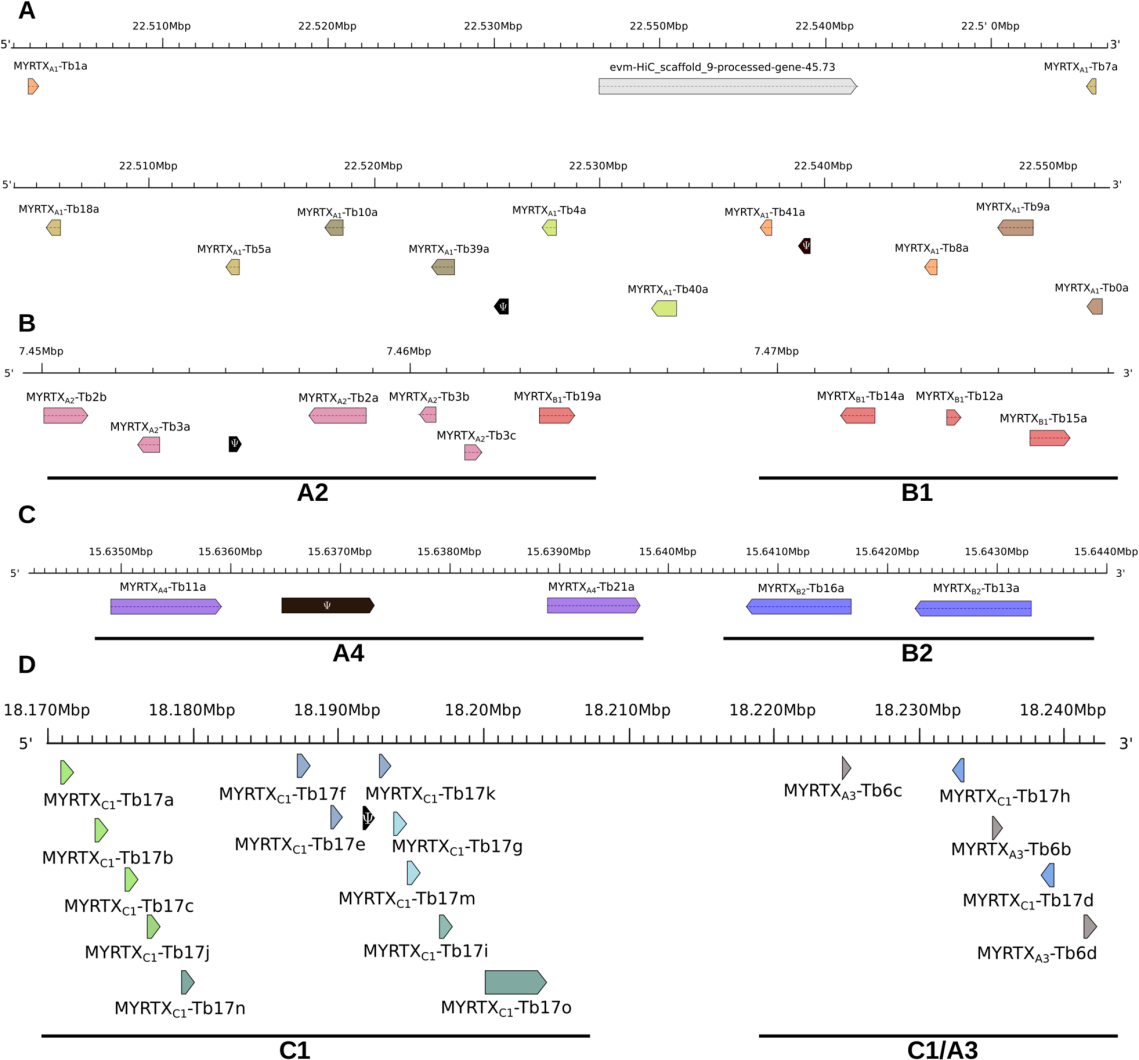
Tandem arrangement of *vpg* on chromosomes. **A**: A1 family gene locus on the chromosome 9, **B**: A2 and B1 families gene loci on the chromosome 8, **C**: A4 and B2 gene loci families on the chromosome 4 and **D**: A3 and C1 families gene loci on the chromosome 6. *MYRTX*_*A1*_*-Tb5b* and *MYRTX*_*A4*_*-Tb11b* are on distinct regions of chromosome 9 and 8 respectively and are not shown. Ψ indicated pseudogenes

To identify the *venom peptide genes (vpg)* of *T. bicarinatum*, we used the previously published proteo-transcriptomic analysis of venom from this species [18]. We blasted the assembled genome with the 37 previously identified venom transcript sequences, resulting in the identification of a total of 44 *vpg* distributed over four different chromosomes (Fig. 1). Throughout this manuscript, venom peptide genes were named according to the nomenclature system described in Additional_file_1, Figure S1 (see Additional_file_2, Table S0 for the correspondence of genes to previously published peptide names). We characterized ten genes encoding additional peptide precursors which were not identified in our previous investigation (Additional_file_1, Figure S2). As these genes are expressed in the venom gland (see paragraph “Expression level of vpg in venom gland cells”), it is likely that they encode peptides secreted in the venom. In the A1 family, three new potential *vpg* were added. Four additional genes *MYRTX*_*A2*_*-Tb2b, MYRTX*_*A3*_*-Tb6d, MYRTX*_*A4*_*-Tb11b, and MYRTX*_*A4*_*-Tb21a* belonging to the A2, A3 and A4 families, respectively, were also found as well as three additional genes in the C1 family (i.e., *MYRTX*_*C1*_*-Tb17m, MYRTX*_*C1*_*-Tb17n, MYRTX*_*C1*_*-Tb17o*) (Fig. 2 and Additional_file_1, Figure S2). We re-annotated *MYRTX*_*A1*_*-Tb18a* gene (Fig. 2 and Figure S5 and S2) and detected a sequence variation in the coding sequence of *MYRTX*_*B1*_*-Tb19a* (Additional_file_1, Figure S2). Genes encoding *MYRTX*_*B1*_*-Tb20a*, *MYRTX*_*A3*_*-Tb6a* and *MYRTX*_*C1*_*-Tb17l* were not found.

Overall, the *vpg* of each precursor family formed clusters organized in tandem repeats on four chromosomes. However, the *vpg* of some precursor families are located on the same chromosome, near to each other (i.e., B1 and A2; A4 and B2) or nested (i.e., C1 and A3). The two *U*_*11*_ genes are on two different chromosomes (i.e., chromosome 4 and 8).

The 13 *vpg* encoding the A1 precursor family are clustered on chromosome 9 and cover a large region of 150 kb (from 22,401,914 to 22,552,361 bp) and an additional region of 550 bp for *MYRTX*_*A1*_*-Tb5b* (from 29,115,914 to 29,116,470 bp). All genes are on the reverse strand except *MYRTX*_*A1*_*-Tb1a* and *MYRTX*_*A1*_*-Tb5b* (Fig. 2A). The intergenic regions were variable in lengths, ranging from 2600 bp to 11,600 bp between most A1 genes but sometimes very extended compared to other families (i.e., 64,000 bp between *MYRTX*_*A1*_*-Tb1a* and *MYRTX*_*A1*_*-Tb7a*, 39,500 bp between *MYRTX*_*A1*_*-Tb7a* and *MYRTX*_*A1*_*-Tb18a)*. The region between the *MYRTX*_*A1*_*-Tb1a* and *MYRTX*_*A1*_*-Tb7a* genes contained a gene encoding an enzyme with serine-type endopeptidase activity *(Corin,* A1Z709, evalue 0*)*, which shares identity with mammalian natriuretic peptide converting enzyme, single-pass transmembrane protein involved in peptide maturation. All other regions contained no other functional genes, but several venom peptide pseudogenes sequences resulting from ancient duplication events. Two regions between the *MYRTX*_*A1*_*-Tb7a* and *MYRTX*_*A1*_*-Tb18a* genes shared sequence identity with two introns of the *MYRTX*_*A1*_*-Tb10a* gene (85% and 78% identity with intron 1 and 91% with intron 2) (data not shown). The sequences following both introns were closely related to the mature sequence of the MYRTX_A1_-Tb10a peptide, but no complete coding region could be identified (data not shown) and no transcriptomic reads were mapped on these loci indicating that none of them contained expressed genes. A third pseudogene was detected between *MYRTX*_*A1*_*-Tb39a* and *MYRTX*_*A1*_*-Tb4a*. *The* ORF displayed a frame shift at the beginning of coding sequence which made the sequence encoding for the signal sequence motif on a different frame than the rest of the coding sequence. This pseudogene is expressed indicating that the production of an mRNA is not impaired. Finally, a fourth pseudogene was detected between *MYRTX*_*A1*_*-Tb41a* and *MYRTX*_*A1*_*-Tb8a*. The start codon is missing in the CDS due to a point mutation that changed the ATG start codon into ATA. Few transcriptomic reads mapped on this locus indicating a weak mRNA production. The TATA boxes of the A1 genes are located at -20 bp of the transcription start sites (TSS) with the following consensus sequence GCTATATAAGCN.

The five genes belonging to the A2 family and the four genes belonging to the B1 family were located in the same region on chromosome 8 from 7,450,038 to 7,461,953 bp for the A2 genes (120 kb) and from 7,463,522 to 7,477,959 bp for the B1 genes (15 kb) (Fig. 2B). For A2 genes, *MYRTX*_*A2*_*-Tb3c* and *MYRTX*_*A2*_*-Tb2a* are on the forward strand while the other three genes are on the reverse strand (Fig. 2B). The intergenic regions ranged from 815 to 5000 bp and do not contain any other genes but two genes predicted as encoding octopamine receptor are located 10 kb upstream *MYRTX*_*A2*_*-Tb2b* and downstream *MYRTX*_*B1*_*-Tb15a* (E1JIT6, evalue 9.90e^−107^ and Q4LBB6, evalue 3.4e^−109^ respectively). An A2 pseudogene corresponding to gene encoding Tb3 peptides was detected in the *MYRTX*_*A2*_*-Tb3a/ MYRTX*_*A2*_*-Tb2a* inter-region. A punctual mutation resulted in a frame shift in the prepro-sequence encoding region. The TATA boxes with (G/A)GTATATAAAGC as consensus sequence are located about -22 bp before the TSS. All B1 genes are on the forward strand, but *MYRTX*_*B1*_*-Tb14a* which is on the reverse strand. The TATA boxes are located -19 bp upstream of the TSS with a consensus sequence of TGTATAAAA(C/T)A. The CDS of *MYRTX*_*B1*_*-Tb19a* does not match the CDS we predicted in our previous study [18] as the mature sequence inferred from the genomic data is ARSRLKIRRMGRK instead of ARSRLKIGRMGR (Additional_file_1, Figure S2).

**Fig. 3:**
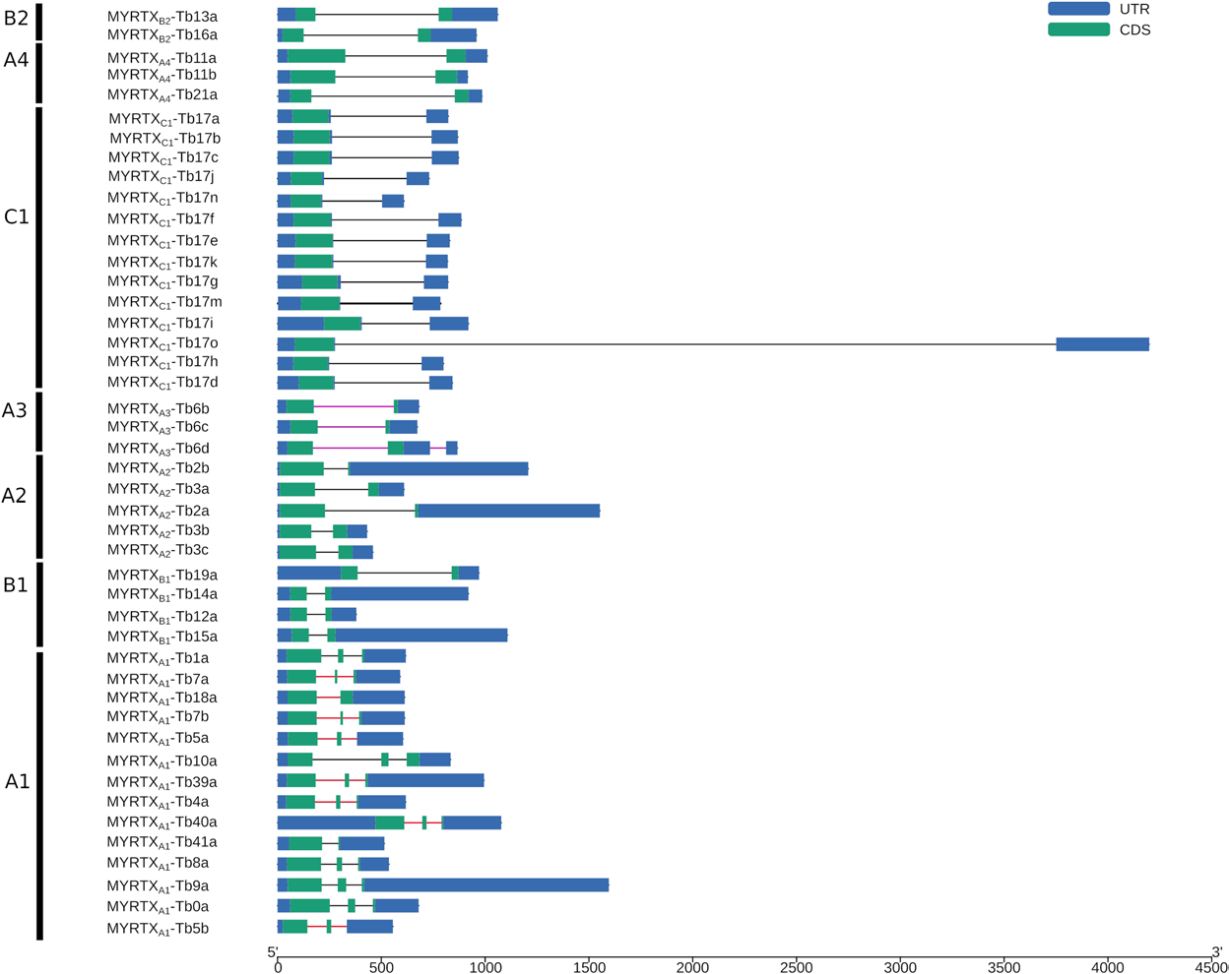
Structural organization of *vpg*. Black lines symbolize introns. Purple and red introns are phase 1 and phase 2 introns respectively

The 15,634,908 to 15,643,312 bp region of chromosome 4 contains both the B2 and *MYRTX*_*A4*_*-Tb11a* genes. The intergenic region between *MYRTX*_*A4*_*-Tb11a* and the two B2 genes is 4760 pb long and only 570 pb separate the two B2 genes (Fig. 3C). A new A4 gene named *MYRTX*_*A4*_*-Tb21a* encoding a predicted mature peptide related to A4 genes previously described in *T. africanum* (U_21_-MYRTX-Ta1a/b), *Manica rubida* (U_20_-MYRTX-Mri1a) and *Myrmica ruginodis* (U_37_-MYRTX-Mru1a) [19, 20] was found between *MYRTX*_*A4*_*-Tb11a* and B2 genes (Additional_file_1, Figure S2 and S3). However, the mature peptide was not detected in our previous studies *T. bicarinatum* venom. An additional A4 pseudogene was also detected near the end of *MYRTX*_*A4*_*-Tb11a*. The ORF lack the ATG and the sequence of prepro-region shares similarity with those of *MYRTX*_*A4*_*-Tb11a/b* precursors, but no mature sequence was detected indicating a premature ending of the ORF. Another predicted gene corresponding to an unknown protein of *D. melanogaster* (Q9VJ69, evalue 9.9e^−101^) was located within the 10 kb upstream *MYRTX*_*B2*_*-Tb13a*. B2 TATA boxes are located at -17 bp (*MYRTX*_*B2*_*-Tb13a*) and -19 bp (*MYRTX*_*B2*_*-Tb16a*) of the TSS and have GGTATAAATTT(G/T) as a consensus sequence. The TATA boxes of *MYRTX*_*A4*_*-Tb11a* and *MYRTX*_*A4*_*-Tb21a (*TCTATAAAAAT and TCTTATAAATC respectively) are located at -22 bp or -19 pb of the TSS.

A new A4 gene, *MYRTX*_*A4*_*-Tb11b,* was located in the 21,187,911 to 21,188,885 bp region of chromosome 8. The coding sequence predicted by maker corresponds to a longer peptide but considering the SRA alignment, the gene ends earlier and does not have a third exon (Additional_file_1, Figure S4). By homology with *MYRTX*_*A4*_*-Tb11a,* we predicted the beginning of mature sequence on the EKE motif (Additional_file_1, Figure S2). The TATA box (ATTATAAATTG) is located -23 bp upstream of the TSS.

Chromosome 6 gathers the fifteen C1 genes as well as the three genes of A3 family at the same locus (Fig. 2D). The C1 genes are arranged in a cluster of thirteen genes between 18,170,944 and 18,200,409 bp on the forward strand and a second cluster of two genes between 18,232,803 and 18,239,159 bp on the reverse strand sharing the same locus with the three A3 genes on the forward strand. The *MYRTX*_*C1*_*-Tb17e/ MYRTX*_*C1*_*-Tb17k* intergenic region contains a *U*_*17*_ pseudogene with a point mutation in the 33rd codon leading to a stop codon and no SRA was mapped to the locus. A short gene is predicted 3 kb upstream *MYRTX*_*C1*_*-Tb17a* but no functional annotation matched its sequence on UniProtKB_refprot/Swiss-Prot database. The 20 kb inter-region between C1 and A3 genes doesn't contain any genes. Three genes are predicted in the 10 kb after *MYRTX*_*A3*_*-Tb6d*. The first is predicted to code an ubiquitin thioesterase otubain-like involved in protein degradation (Q9VL00, evalue 1e^−116^), the second has no predicted function and the third as a BLOC-1-related complex subunit 7 (lysosomal protein) (A1ZBV5, evalue 1.5e^−23^).

The predictive TATA box showed (A/T)(A/G)TATAAAAG as consensus sequence and was located -22 bp before the TSS. For *MYRTX*_*C1*_*-Tb17o*, the first TATA box motif (TATATAAAAA) is located -120 bp upstream of the TSS.

### Genetic structure of venom peptide encoding genes

A1 genes have canonical (GT/AG) introns and all genes displayed two introns except for *MYRTX*_*A1*_*-Tb41a* which is a single intron gene (Fig. 3). Alignment of A1 genes reveal that *MYRTX*_*A1*_*-Tb41a* intron corresponded to intron 2 of other A1 genes and that intron 1 and exon 2 sequences were missing explaining the predicted mature peptide very short length (Additional_file_1, Figure S2). The first introns of the A1 genes encoding disulfide-bonded mature peptides are all phase 2 (see Figure S5). For *MYRTX*_*A1*_*-Tb18a*, an alternative splicing site in the second exon processes the gene into two different transcripts (Additional_file_1, Figure S5). A first transcript, with intron 2 retention, encoded a two disulfide-bonded peptide (i.e., Tb18a), previously reported by the transcriptomic data but not confirmed by mass spectrometry and a second transcript encoding a peptide with a single disulfide bond (Tb7b) which was detected in the mass spectrometry analysis (Additional_file_1, Figure S2). It should be noted that a Tb7b homologue was also identified and confirmed in *T. africanum* venom (U_7_-MYRTX-Ta1b, DVNCEITPFHPKCRGVAP) [19], suggesting that this is the mature form in the venom and that the two disulfide-bonded peptide ORF is an aberrant splicing profile that is probably non-translated. No peptide with two intrachain disulfide bonds has been moreover found in *Tetramorium* venom, nor in other Myrmicinae venom [19].

The first exons of the A1 genes encompass the entire prepro-sequence and the beginning of the mature peptide, while the second exons are short, averaging 26 bp in length (from 13 to 42 bp). For *MYRTX*_*A1*_*-Tb5a* and *MYRTX*_*A1*_*-Tb5b*, the second exons encoded the end of the mature peptide sequence. The third exon is then untranslated. For the other A1 genes, the third exons contain short CDS (ranging from 9 to 27 bp) that include the “GKK” amidation signal for several toxins (*MYRTX*_*A1*_*-Tb0a, MYRTX*_*A1*_*-Tb1a, MYRTX*_*A1*_*-Tb8a, MYRTX*_*A1*_*-Tb9a* and *MYRTX*_*A1*_*-Tb4a*).

All others *vpg* are single intron genes with canonical splicing sites (GT/AG) (Fig. 3), except for the *MYRTX*_*A2*_*-Tb3a* intron that displays non-canonical GC/AG splicing sites (Additional_file_1, Figure S6) at the same position of the ORF as the other *MYRTX*_*A2*_*-Tb3b/c* genes (i. e. after the sequence encoding the LL motif of the mature sequence). The first exons of the C1 genes (282 bp in mean) contain the entire ORF followed by a canonical intron of 446 bp in mean starting one to four codons after the stop codon and a second untranslated exon. The *MYRTX*_*C1*_*-Tb17o* intron is particularly extended with a length of 3473 bp (Fig. 3). The introns of all other single intron genes are found in the middle of the CDS encoding the mature sequences. The A3 genes that code for peptides with a disulfide bond share phase-1 introns (Fig. 3). According to the SRA alignment data, *MYRTX*_*A3*_*-Tb6d* has a third untranslated exon (Additional_file_1, Figure S7). Only *MYRTX*_*A4*_*-Tb11a/b* genes have phase-0 introns among genes encoding disulfide-bonded peptides. Phase-1 or -2 introns are indeed specific features of A1 and A3 genes that encode disulfide-bonded peptides.

### Evolution profiles

We compared the coding and non-coding sequences of *vpg* and determined percentage identity (Id%) within each family (Additional_file_2, Table S1).

The percentage of sequence identity of *vpg* varies between different regions. Promoter sequences are the least conserved regions with the lowest identity percentage (Id%) for *MYRTX*_*A4*_*-Tb11a/b*, C1 and A1 genes (46%, 47% and 48% respectively). However, when we examined alongside the promoter region of these three families (Additional_file_2, Table S2) the Id% reached 64% and 66% within the -100 pb for A1 and *MYRTX*_*A4*_*-Tb11a/b* promoters, respectively. The Id% of the C1 family remain low throughout the length of the promoter region. In addition, some genes within a family shared very similar sequences but have divergent promoters, such as *MYRTX*_*B2*_*-Tb13a* and *MYRTX*_*B2*_*-Tb13a* (85% gene identity versus 52% promoter identity), *MYRTX*_*A1*_*-Tb7a/ MYRTX*_*A1*_*-Tb18a* (90% gene identity versus 38% promoter identity), *MYRTX*_*C1*_*-Tb17k*/*MYRTX*_*C1*_*-Tb17e/MYRTX*_*C1*_*-Tb17f* (90% gene identity versus 45% promoter identity), *MYRTX*_*C1*_*-Tb17b* and *MYRTX*_*C1*_*-Tb17c* (94% vs 51%) and *MYRTX*_*A2*_*-Tb2a/ MYRTX*_*A2*_*-Tb2b* (86% gene identity and 45% promoter identity). In a few cases of neighbouring *vpg* however, promoter sequences are as well conserved as gene sequence *MYRTX*_*C1*_*-Tb17h*/ *MYRTX*_*C1*_*-Tb17d* (87%/94%) or more conserved for *MYRTX*_*A3*_*-Tb6b/ MYRTX*_*A3*_*-Tb6c* (97%/72%), *MYRTX*_*C1*_*-Tb17j*/ *MYRTX*_*C1*_*-Tb17c* (91%/70%).

**Fig. 4:**
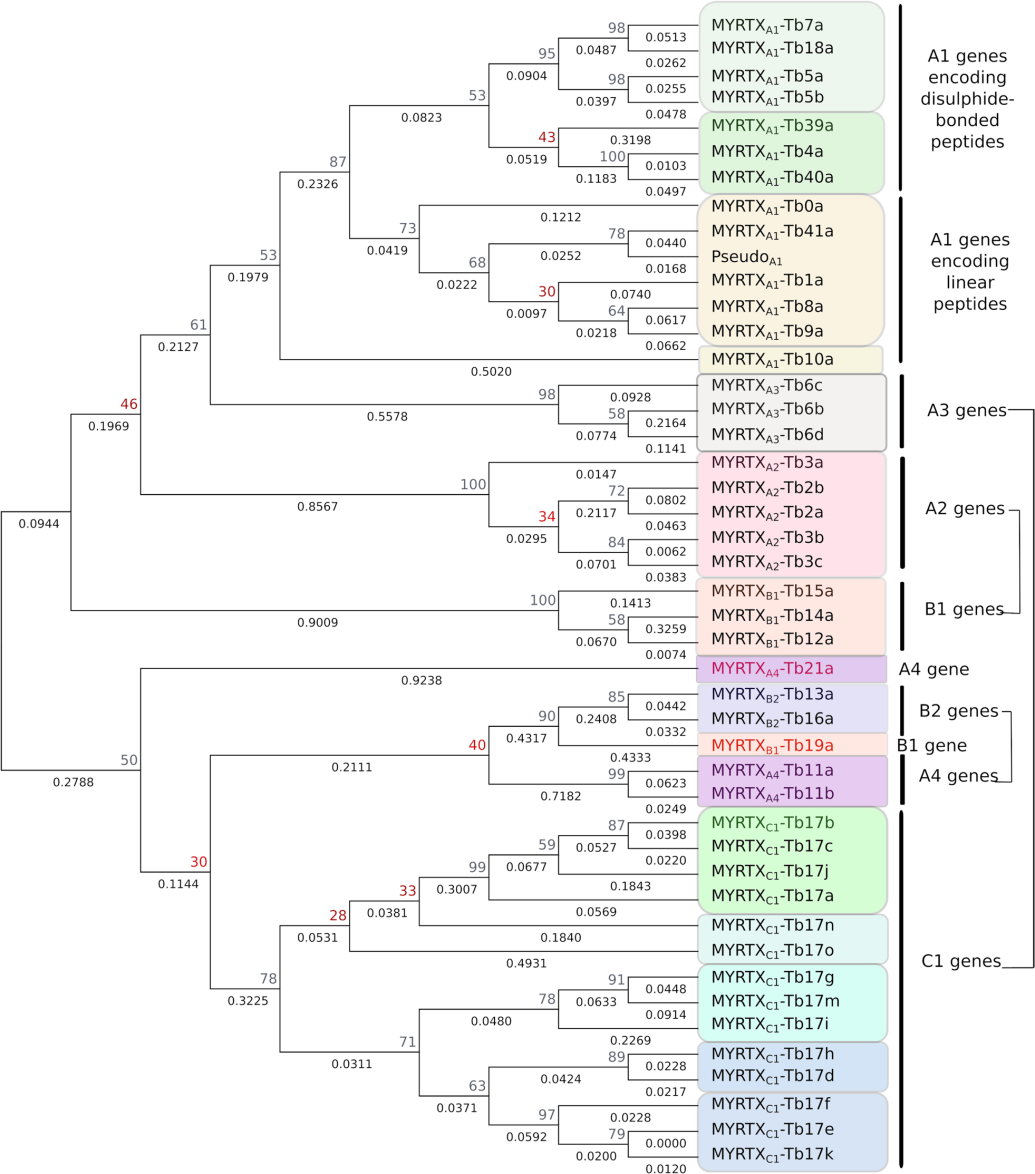
Evolutionary analysis by Maximum Likelihood method of *vpg* genes sequences. The evolutionary history was inferred by using the Maximum Likelihood method and Tamura 3-parameter model. The tree with the highest log likelihood (-5038.21) is shown. The percentage of trees in which the associated taxa clustered together is shown next to the branches. Initial tree(s) for the heuristic search were obtained automatically by applying Neighbor-Join and BioNJ algorithms to a matrix of pairwise distances estimated using the Tamura 3 parameter model, and then selecting the topology with superior log likelihood value. A discrete Gamma distribution was used to model evolutionary rate differences among sites (5 categories (+ *G*, parameter = 5.4840)). This analysis involved 45 nucleotide sequences. All positions with less than 95% site coverage were eliminated, i.e., fewer than 5% alignment gaps, missing data, and ambiguous bases were allowed at any position (partial deletion option). There was a total of 177 positions in the final dataset. Evolutionary analyses were conducted in MEGA11. Pseudo_A1_ correspond to the pseudogene located between *MYRTX*_*A1*_*-Tb39a* and *MYRTX*_*A1*_*-Tb4a*

We performed a ML phylogenetic analysis of *vpg* sequences (Fig. 4). *Vpg* clustered according to the precursors families with two subgroups gathering A1, A2, A3 and B1 genes on one side and C1, A4 and B2 on the other. A1 genes encoding linear peptides gathered on the same branch as well as A1 genes encoding disulphide-bonded peptides with two distinct subgroups one gathering *MYRTX*_*A1*_*-Tb5a, MYRTX*_*A1*_*-Tb5b, MYRTX*_*A1*_*-Tb7a* and *MYRTX*_*A1*_*-Tb18a* and the other *MYRTX*_*A1*_*-Tb4a, MYRTX*_*A1*_*-Tb39a and MYRTX*_*A1*_*-Tb40a*. *MYRTX*_*A1*_*-Tb10a* remained aside. A1 genes that are closely located on the chromosome are not necessarily close in the tree. In contrast, the closer the C1 genes are on the chromosome, the closer they are on the tree. *MYRTX*_*C1*_*-Tb17d* and *MYRTX*_*C1*_*-Tb17h* which are distant from the rest of C1 genes appear to be related to *MYRTX*_*C1*_*-Tb17k*, *e* and *f* cluster. *MYRTX*_*C1*_*-Tb17o* and *MYRTX*_*C1*_*-Tb17n* are alone on their respective branch*.* C1 and A3 and A2 and B1 genes cluster on different branches despite their location on the same chromosomal region. *MYRTX*_*A4*_*-Tb11a/b* and B2 genes clustered on same branch along with *MYRTX*_*B1*_*-Tb19a*. *MYRTX*_*A4*_*-Tb21a,* despite its location between *MYRTX*_*A4*_*-Tb11a* and B_2_ genes, is alone on its branch. As some main branches of the cluster A4, B2 and C1 are supported by weak bootstrap percentages probably because of the sequence diversity of the alignment, we performed a ML analysis with a sub-alignment of A4, B2, C1 and B2 genes (Additional_file_1, Figure S8). Here, percentages were all above 70%. C1 genes always clustered according to their chromosomal position, U_11_ and B2 genes were on the same branch but *MYRTX*_*B1*_*-Tb19a* gathered this time with B1 genes and *MYRTX*_*A4*_*-Tb21a* was much more related to A4/B2 cluster. We also performed the same analysis for the subgroup gathering A1/A2/A3/B1 (Additional_file_1, Figure S9). A1 *vpg* showed the same clustering, A2 *vpg* were still in the same main branch as A1 while A3 *vpg* clustered this time closer to B1 family than A1 family. In general, percentages were higher on each node.

### Expression and potential regulation of *vpg*

#### Expression level of vpg in venom gland cells

*T. bicarinatum* genome allowed us to re-analyse previously published datasets. Reads from the venom gland or whole abdomen transcriptomic data available in Genbank were mapped to this genome to accurately evaluate the expression of the genes. All 44 *vpg* identified in the genome were expressed in the venom glands, accounting for 88% of the total TPM in the venom glands transcriptome.

**Fig. 5:**
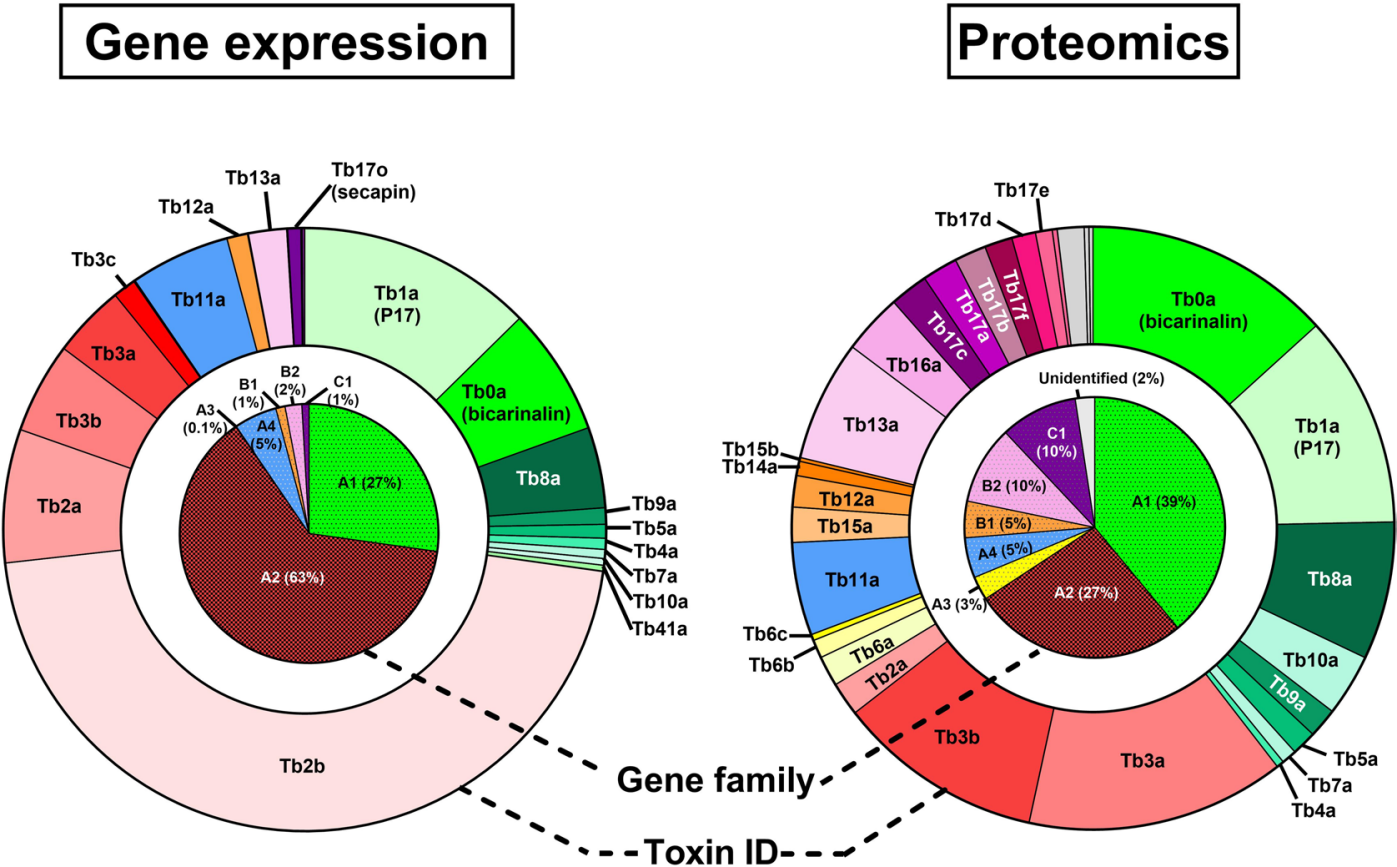
Proportion of toxins in *T. bicarinatum* venom assessed by transcriptomics and proteomics. Proteomic data are from [18]

Aculeatoxin-related genes (i.e., A1, A2, A3, A4) are highly expressed in *T. bicarinatum* venom accounting for 95% of the relative expression of all genes encoding venom peptides. The high proportion of aculeatoxin-derived peptides in the venom is confirmed as they represent almost 75% of the total toxins previously identified by the proteomics analysis (Fig. 5). Among the aculeatoxins*,* the A2 genes are the most expressed accounting for 63% of the 44 expressed *vpg* (Fig. 5 and Additional_file_2, Table S3). *MYRTX*_*A2*_*-Tb2b* is a highly expressed *vpg* encoding a decapeptide which have not been found in the previous proteo-transcriptomic investigations of *T. bicarinatum* venom. However, as mentioned above, both *MYRTX*_*A2*_*-Tb2a/b* genes exhibit a rather unusual hit pattern as the most frequent hits are mapped in the 3’UTR region (Additional_file_1, Figure S10). The CDS is therefore under-expressed compared to the 3’UTR and leads to an overestimation of the expression of the *MYRTX*_*A2*_*-Tb2a/b* genes. This is confirmed by the proteomic data which clearly showed that the proportion of Tb2a/b peptides is much lower and that the Tb2b peptide was not detected in the venom. In the A1 family, the *MYRTX*_*A1*_*-Tb5b* (2.5 TPM) is almost 3000 times less expressed than the *MYRTX*_*A1*_*-Tb5a* (7300 TPM) even though these two genes share the same coding and promoter sequences. *MYRTX*_*A1*_*-Tb5b* is located near the end of chromosome 9. This region contains only six additional genes downstream of *MYRTX*_*A1*_*-Tb5b* and the closest gene upstream *MYRTX*_*A1*_*-Tb5b* is located at 720 kb. All these genes are not or hardly expressed suggesting that *MYRTX*_*A1*_*-Tb5b* locus is likely in a zone of poor chromatin accessibility.

The A1 genes expression is dominated by two genes coding Tb0a (bicarinalin) and Tb1a (P17) which are a membrane-disrupting peptide [21] and MRGPRX2-GPCR agonist [22], respectively. Linear peptides are tenfold more expressed than disulfide-bonded peptides. The novel venom peptides identified in this study and *MYRTX*_*A1*_*-Tb18a* are the least expressed *vpg*, which may explain why their corresponding mature peptides were not detected in previous MS analysis. The level of expression of the A4, B1, B2 and C1 genes also vary considerably with one gene being much more expressed than its counterparts in each family. Although the *MYRTX*_*C1*_*-Tb17o* gene was not reported in our previous study, it is the most expressed C1 gene with 6000 TPM. *MYRTX*_*B1*_*-Tb12a*, *MYRTX*_*B2*_*-Tb13a, MYRTX*_*A3*_*-Tb6b* and *MYRTX*_*A4*_*-Tb11a* are the most expressed genes in B1, B2, A3 and A4 families, respectively. *MYRTX*_*A4*_*-Tb21a* and *MYRTX*_*B1*_*-Tb19a* are among the least expressed genes, which may explain why we did not detect the corresponding mature peptides in *T. bicarinatum* venom. Interestingly some neighbouring genes share similar expression level while having divergent promoter as *MYRTX*_*C1*_*-Tb17b/c* or the opposite as *MYRTX*_*C1*_*-Tb17c/j and MYRTX*_*A3*_*-Tb6b/d*.

#### Prediction of regulatory mechanisms of vpg expression

To understand regulatory mechanisms involved in the heterogeneous *vpg* expression, we analysed the binding sites (BS) for transcription factors (TF) coded by genes which were highly expressed in venom glands.

Among the 455 genes expressed with more than 40 TPM (*vpg* excluded) in the *T. bicarinatum* venom gland, 376 were functionally annotated with an evalue > 10^–10^ and 16 corresponded to DNA-linked transcription factors (GO molecular function: GO:0003700, GO:0000981, GO:0000978) (Additional_file_2, Table S4). We selected seven TFs (i.e., DimmED, CrebA, GATA-Srp, GATA-Pnr, Stat92E, Ken, Cnc). DimmED and CrebA (*Cyclic-AMP response element binding protein A*) are of particular interest as they encode TF involved in the control of canonical secretory pathways genes [23, 24]. These two factors are 8.5 and 6.8 times more expressed in venom glands than in the rest of the ant, suggesting a role in the venom secretory pathways. The GATA TFs, serpent (Srp) and pannier (Pnr), were also more highly expressed in venom glands than in the rest of the body. Interestingly *Drosophila* Srp is required for the development of fat body but also for the tissue-specific immune response in *Drosophila* larvae [25] and adult [26, 27]. GATA BS are located in the promoter of genes encoding *Drosophila* Host defence peptides (HDPs) which are small secreted peptides with antimicrobial function and GATA TFs are required in several tissues to induce HDP genes expression [26, 28]. The TFs Stat92E (*Signal Transducer and Activator of Transcription 92E*) and Ken were also included in the analysis although they did not show an overexpression in venom gland tissues. They are involved in JAK/STAT pathway which controls many biological processes, including the immune response [29]. Besides, Stat92E positively regulates Pnr [30]. Finally, we selected the Cap'n'collar (Cnc) proteins, which control genes involved in the protection against oxidative stress [31] for their extensive expression in venom gland compared to the rest of the body.

**Fig. 6:**
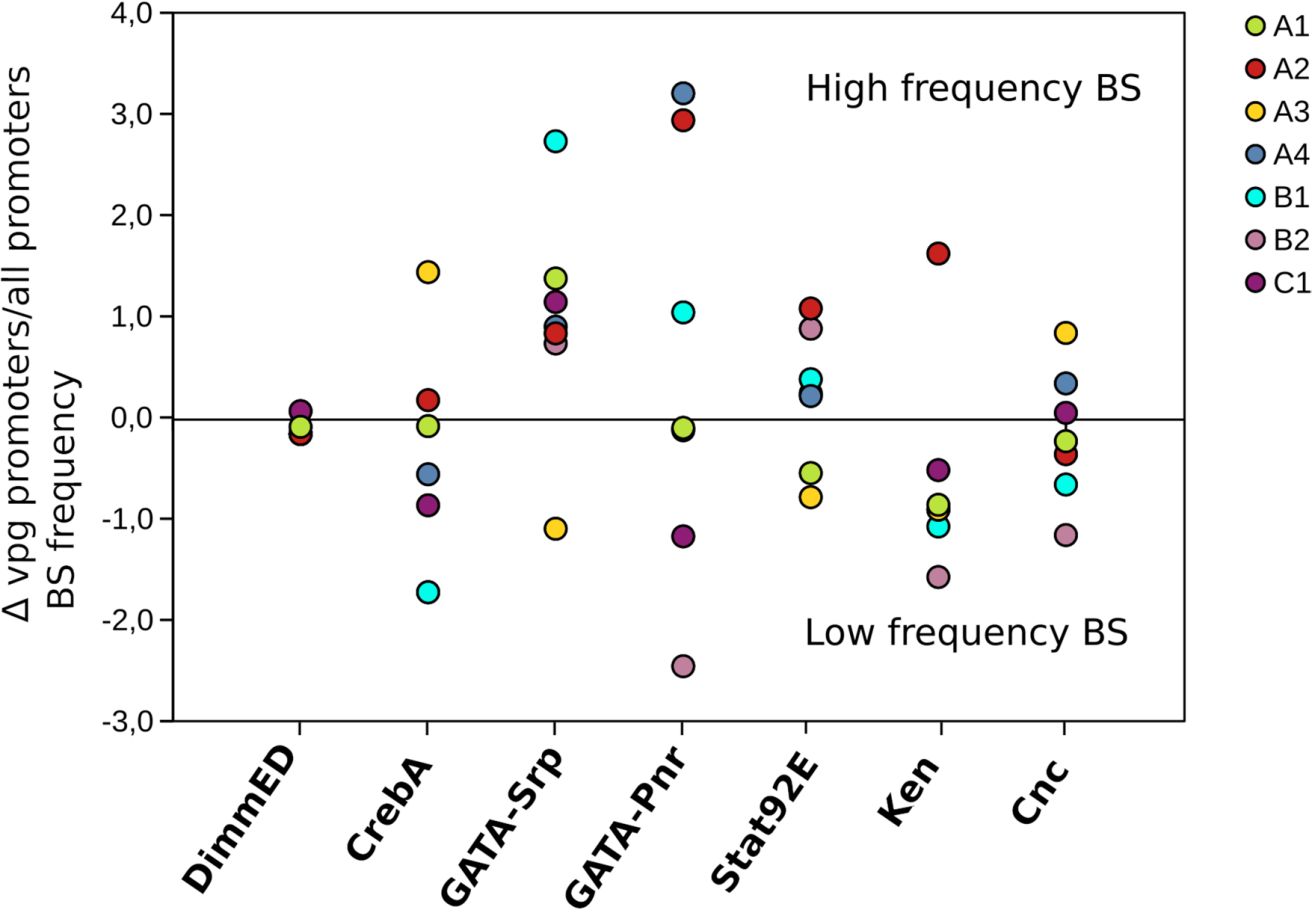
Frequencies of TFBS on *vpg* promoters compared to frequencies on promoters of all *T. bicarinatum* genes. Values on Y axis correspond to the difference between TFBS frequency on vpg promoters and TFBS frequency on all *T. bicarinatum* gene promoters. For Cnc only 500 pb among the TSS have been considered (see M&M for explanations)

We then searched for the binding sites (BS) of selected TFs on both the promoter (-1000 pb among TSS) (Additional_file_1, Figure S11) and intron1 sequences of the *vpg* using *Drosophila* matrices (see detail of validation in Additional_file_3)*.* We compared the BS frequency of each TF found in *vpg* promoters with the frequency (called random frequency) in the promoters of other *T. bicarinatum* genes as control. (Additional_file_2, Table S5 and Fig. 6).

DimmED is probably not involved in the control of *vpg* expression since no or very few BS were found in the *vpg* promoters (Fig. 6) (1000 bp upon the TSS) or in intron 1 (data not shown). The strong overexpression of the DimmED gene observed in the venom glands would rather be related to its function in the control of the general secretory machinery. By contrast, the analysis revealed a hotspot of GATA TF Srp BS with a mean frequency higher than the random frequency for all families except A3. The frequency of GATA TF Pnr BS is also above the random frequency for A2, A4 and B1 families (Fig. 6 and Additional_file_2, Table S5). Many Srp BS are in the proximal region (-400 bp above the TSS) of several A1 genes (i.e., *MYRTX*_*A1*_*-Tb10a, MYRTX*_*A1*_*-Tb4a, MYRTX*_*A1*_*-Tb18a, MYRTX*_*A1*_*-Tb39a* and *MYRTX*_*A1*_*-Tb0a*) yet having few sequence identity (52%) (Additional_file_1, Figure S11). In the promoter sequence of *MYRTX*_*A1*_*-Tb0a,* Srp binding sites were almost the only sites that were detected. Furthermore, Amadeus predicted an enriched motif in the A1 proximal promoter sequences (-400 pb) with similarity to GATA BS (Human GATA3 and Drosophila Srp) indicating that GATA motifs are widely distributed in A1 proximal promoters (Additional_file_2, Table S6). For some C1 genes among the most expressed, a region between -900 and -500 pb among the TSS is particularly rich in Srp BS (10, 6, 4 and 6 for *MYRTX*_*C1*_*-Tb17a*, *MYRTX*_*C1*_*-Tb17f*, *MYRTX*_*C1*_*-Tb17e* and *MYRTX*_*C1*_*-Tb17g* respectively) (Additional_file_1, Figure S11). Amadeus analysis of promoters between -1000 and -400 pb reveals three enriched motifs (Length 12) that could correspond to GATA BS, but annotation scores were not as good as those observed for A1 and A2 proximal promoter regions (data not shown). Introns of some families also contain GATA BS as A4, C1 and B2 introns (data not shown). For A3 genes, a conserved motif of Pnr and CrebA BS localized to the promoter proximal regions (Additional_file_1, Figure S11) but no enriched motifs were predicted with Amadeus. In A3 promoters, Cnc BS were also found with a frequency higher than the random frequency in the 500 bp among the TSS. A2 gene promoters showed the most complex BS pattern with Srp, Pnr, Stat92E, and Ken TF BS frequencies higher than the random frequency (Fig. 6). A conserved pattern of CRE (cis-regulatory elements) for Pnr/ Srp and Stat92E was present in the proximal region of A2 promoters (-250 bp from the TSS) (Additional_file_1, Figure S11). Amadeus analysis also revealed an enriched motif corresponding to GATA sites (human GATA3 and Srp BS) in the proximal promoter (-400 pb among TSS). Moreover, position on A2 promoter of the enriched motif 1 correspond to that of distal sequence of the first Stat92E BS (Additional_file_2, Table S7**)**.

## Discussion

### The diversification of vpg paralogs is driven by tandem duplication of a common ancestor

The global *vpg* organization is consistent with a common ancestor in each precursor family and diversification by local tandem duplication. Genomic studies in other venomous animals such as snake [32, 33], sea anemones [34], spider [35], cone [36], and Apidae [10] showed also tandem duplication of some genes coding venom peptide toxins. The number of paralogous genes within A1 and C1 families may indicate an ancient origin of these *vpg* or a high frequency of duplication. However, our results revealed that *vpg* of some precursor families are located on the same chromosome which raises the question of a possible common ancestor for several families. Also, *Tb11* and B2 genes still gather on ML tree suggesting that they may have derived from a common ancestor. In contrast, all other *vpg,* even those that are located close together in the genome*,* were clustered on different branches according to their family suggesting that most of *vpg* family may have originated from different ancestral genes. The position of some *vpg* remains uncertain such as *MYRTX*_*A4*_*-Tb21a* which gathered beside or within A4/B2 cluster depending on the sequence alignment that was considered in the ML analysis. This suggest that the A4 family might have to be reconsidered. As mentioned above, venom peptide precursors sharing high id% with Tb21a in their prepro-sequences (Additional_file_1, Figure S3) (88 Id% between both Tb21a and U_21_-MYRTX-Ta1a prepro-sequences, 65 Id% between *MYRTX*_*A4*_*-Tb21a* and other A4 genes) were found in other Myrmicinae species [19]. The analysis of the sequences of genes that encoded these precursors would allow to know if they would cluster with B2 genes, with *MYRTX*_*A4*_*-Tb11a/b* genes or if they would constitute together a novel family. We also noticed that A1 *vpg* did not cluster on the tree according to their position on the chromosome suggesting that long-distance duplication events may have occurred during the evolution of family A1 genes. This might explain why intergenic regions are particularly long in this family. On the other hand, the closer the C1 genes are on the chromosome, the closer they are on the tree indicating evolution by successive short-distance duplication. The venom profile of a closely related ant species, *T. africanum*, was previously reported and provides a glimpse into the evolution of venom peptide profiles in *Tetramorium* ants. The venom of *T. africanum* indeed contains homologues of Tb1a, Tb0a, Tb4a, Tb7a and Tb7b peptides that may be encoded by orthologs of their corresponding genes but no homologue of Tb5a, Tb8a and Tb9a were found [19] suggesting that the duplication leading to *MYRTX*_*A1*_*-Tb5a* or to *MYRTX*_*A1*_*-Tb8a* and *MYRTX*_*A1*_*-Tb9a* may have occurred specifically in *T. bicarinatum* genome. By contrast, we found three disulfide-bonded peptides in *T. africanum* as well as one linear peptide with no equivalent in *T. bicarinatum* venom.

The organization of *vpg* introns/exons has been conserved over the course of repetitive gene duplication events and all genes except *MYRTX*_*A2*_*-Tb3a* have canonical splicing sites. However, we noted specific shifted introns for all genes encoding disulfide-bonded peptides in the A1 and A3 family suggesting a putative mechanism based on the control of RNA maturation to drive the production of disulfide-bonded peptides in *Tetramorium* venoms. Moreover, the phylogenetic tree of the A superfamily showed that A1 *vpg* encoding disulfide-bonded peptides were clustered on the same branch suggesting a common primary ancestor for A1 genes encoding disulfide-bonded peptides.

For *MYRTX*_*A2*_*-Tb3a*, the non-canonical 5’ site is at the same location relative to the CDS as for *MYRTX*_*A2*_*-Tb3b/c* genes. Preservation of the 5' splice site position by recruitment of a non-canonical splice motif may have contributed to the preservation of the function of the *MYRTX*_*A2*_*-Tb3a* paralog. Furthermore, we have previously shown that the position just after this 5’ splicing site is subject to pervasive positive selection [19] and therefore might be related to Tb3a/b/c molecular neofunctionalization. This peptide family has recently been shown to play a defensive role by modulating voltage-gated sodium channels (Na_V_) in vertebrates [37] and such neofunctionalization could be associated with fine-tuning the toxins affinity with Na_V_ receptor of different type of predators.

### The regulation of *vpg* paralogs in the venom gland results in high but intrafamily variable expression levels

Almost all *vpg* exhibited high expression levels indicating that all paralogs are selected to ensure production of peptides in large amounts in the venom. However, within the *vpg* families, one gene or a couple of genes are always much more strongly expressed than the others. For instance, in the A1 family, genes encoding linear peptides are more highly expressed that those encoding disulfide-bonded peptides. The driving forces for gene retention might thus be based on both neofunctionalization at molecular level and acquisition of a required expression level, ultimately leading to a cocktail of toxins with multiple biological activity and in variable proportion. The expression of human paralogs has been investigated and some studies have demonstrated that older paralogs tend to have higher expression than youngest derived ones [38, 39]. It has also been proposed that copies evolving toward lower expression are fixed to maintain balance of genetic expression after duplication [39]. These studies suggest that genes with the highest expression level are the oldest, and thus that the less expressed genes are the youngest which is consistent with our observations for the A1 family where *vpg* encoding disulfide-bonded peptides may be more recently derived than those encoding A1 linear peptides*.* To date A1 disulfide-bonded peptides have not been identified in the venom of Myrmicinae other than *Tetramorium* suggesting that these peptides are recent in the evolutionary history of the Myrmicinae ants. In addition, comparison of the expression of primate orthologous genes showed that ancestral paralogs exhibited also more conserved patterns of expression than derived paralogs [38]. Our previous published data on *T. africanum* showed that transcripts coding Tb0a and Tb1a homologs are highly represented in the venom gland transcriptome (84,000 and 17,000 TPM, respectively) and that their corresponding mature peptides were abundant in the venom. On the opposite, transcripts encoding A1 disulfide-bonded peptides are less abundant (6500 TPM for transcript coding for U_7_-MYRTX-Ta1a and 3200 for U_4_-MYRTX-Ta1a) as their corresponding mature peptides in the venom. It appeared that the expression pattern of putative orthologs is conserved between these two species, but it is not the case for all genes. For instance, U_21_-MYRTX-Ta1a is among the most abundant peptide in *T. africanum* venom while Tb21a is much less expressed in *T. bicarinatum* venom gland and the mature peptide is undetectable in the venom. Evolutionary mechanisms leading to *vpg* expression levels are therefore more complex than the conservation of putative orthologous expression pattern between different species. Nevertheless, quantification of *T. africanum* transcripts is only estimated as some contigs have multiple or partial ORFs. The analysis was derived from a de novo assembly which did not allow us to obtain the exact sequence of the transcripts and their expression levels. To study the precise orthologous expression levels, it would be relevant to perform further genomic and transcriptomic investigations with several other *Tetramorium* species to identify orthologous genes as well as their expression profile. This would provide information on gene evolutionary patterns to advance the understanding of the history of duplication events that led to the diversity of venom genes.

Furthermore, we found that both *MYRTX*_*A2*_*-Tb2a/b* genes exhibited an unusual expression pattern with the long 3’UTRs sequence being much more highly represented among transcriptomic reads than the CDS sequence. Such a long 3'UTR was also observed in the transcript encoding U_2_-MYRTX-Ta1a in the venom of *T. africanum* [19] which shared 86% of sequence identity with both *MYRTX*_*A2*_*-Tb2a/b* 3’ UTRs from *T. bicarinatum*. Similar to *T. bicarinatum*, the U_2_-MYRTX-Ta1a encoding contigs were also highly represented in the transcriptome (73 and 50 000 TPM) while the relative abundance of the mature peptide is less than 1% of the venom in *T. africanum* venom. Few studies have investigated such a differential expression CDS/3’UTR, but it has been reported in some mouse development genes [40, 41] while no regulatory mechanisms or functions have been identified. It demonstrated the relevance of genomic approaches to study the function of venom peptides and raises the question of whether the selection of this control of *MYRTX*_*A2*_*-Tb2a/b* gene expression is related to their biological function. To date no function has been assigned to these peptides. Tb2a/b peptides have only been detected in *Tetramorium* venom [19] which may indicate that *MYRTX*_*A2*_*-Tb2a/b* genes are not expressed or absent in other Myrmicinae.

### The *vpg *regulatory network may be driven by GATA factors: inference about *vpg* recruitment

Analysis of the *vpg* promoter (-1000 pb among the TSS) revealed that they shared only weak sequence identity even for the neighbouring genes suggesting that the duplication events rarely include enhancer sequences and that neighbouring 5’ sequences would have been recruited as enhancers instead. However, how the above-mentioned regulatory patterns have been fixed remains to be determined. Our data showed that GATA TF had a high BS frequency in all families suggesting their putative role in controlling the general *vpg* expression. However, a general intrafamily conserved CRE pattern is difficult to highlight except for A2 family proximal promoters due to the poor sequence identity. The enrichment of GATA-BS in most of the promoters could indicate that the surrounding regions of fixed *vpg* have acquired or already exhibited GATA BS hotspots that contribute to the preservation of functional copies. GATA BS were scarce in A3 promoters suggesting other regulation pathways. As previously mentioned, *Drosophila* GATA TFs control many genes involved in immune processes particularly those encoding HDPs and some are organized in tandem repeat as *cecropin*, *drosomycin-like* and *attacin* genes. It also has been demonstrated that the 20-Hydroxyecdysone signalling leading to the expression of some immune genes is dependant of Pnr and Srp TF [27]. While no *Drosophila* HDP homologues have been found in *T. bicarinatum* genome, some peptides (i.e., Tb1a, Tb9a and Tb0a) of *T. bicarinatum* venom exhibit biological functions similar to HDP (i.e., cytotoxic activity against bacteria or modulation of GPCR involved in mammalian innate immune system) [21, 22, 42]. Moreover, C1 genes encode for Secapin-like peptides and, among reported Secapin-like peptides, AcSecapin-1 from the bee venom, exhibit an antimicrobial activity and its expression is upregulated in fat body after bacterial challenge [43]. We hypothesize that some venom peptides may play a role in ant immunity, which raises further questions about the function of ancestral *vpg* genes and how *vpg* have been recruited and fixed in venom glands after/before segmental duplications. Venom has been suggested to participate in social immunity in some hymenopteran such as bees [44] and wasp [45], but to date no evidence except for bee secapin linked venom function to individual innate immunity. Several models have been proposed to explain recruitment and fixation of genes after duplication event: neofunctionalization and subfunctionalization. Neofunctionalization is mainly based on the acquisition of a new function by duplicated copies through mutations in the ORF. Ancestral and derived paralogous genes may share spatial or temporal expression. Subfunctionalization is based more on the acquisition of a new spatial or temporal expression pattern by duplicated copies through mutations in enhancer sequences; ancestors and paralogous genes may share the same function. [46]. Paralogous genes can also be preserved with a functional and/or expression redundancy particularly if they are organized in tandem repeats [39]. For venom genes fixation, these mechanisms have been extensively debated [47]. In snake venom, both neofunctionalization and subfunctionalization have been thus proposed for some toxin genes [33, 48, 49]. Two gene families encoding snake venom peptides have been proposed to be fixed after subfunctionalization, myotoxin (crotamine) genes that may be derived from neighbouring non-toxic β-defensin-like peptides and NGF-β (Nerve Growth Factor β polypeptide) which may be derived from non-toxic form of NGF [33, 50]. In both cases, ancestral genes are expressed in non-venomous tissues. In house spider, *latrodectin* genes may be derived from the neighbouring CHH/IPT (crustacean hyperglycemic hormone/insect ion transport peptide) neuropeptide superfamily with which they share sequence homology [35]. In parasitoid wasps, some venom genes have been also co-opted from existing genes expressed in other tissues [9, 51]. Analysis of the *T. bicarinatum* genome revealed no genes in the vicinity of the *vpg* loci that share sequence identity with *vpg* and are involved in other functions. A defensin gene has been identified in the *T. bicarinatum* genome but was localized on chromosome 7 and has no obvious sequence identity (~ 20%) with other *vpg*. The difficulty in finding putative ancestral genes near the *vpg* suggest that the original genes are already organized to encode secreted peptides having similar activity as the venom peptides and may be involved in the "endo" defense function of the organism through a specific expression in non-venomous tissues, such as in the fat body. Ants, one of the most highly derived hymenopterans, have likely inherited this characteristic from an ancient hymenopteran ancestor. An ecological shift during the evolution of hymenopteran may have led to the weaponization of some non-venomous genes through their recruitment/overexpression in exocrine tissues such as sexual accessory gland of female from which hymenopteran venomous apparatus derived [4] to gain an exochemical function. Additional segmental duplications and mutations of copies then led to a diversification/optimization of venom chemical arsenal. The apparition of parasitoïdism which occurred early during hymenopteran evolution may also have triggered the weaponization of some genes, possibly belonging to the immune system, as one of the main goals of parasitoid wasp venoms is to evade the host's immune system [52].

 Inter-regions or more distant regions might also be involved in the *vpg* regulation. Shew et al. examined the activity of promoter or more distant enhancer sequences of ancestral and derived paralogs [38]. They found no unique pattern, as differential regulation sometimes involved distant enhancer sequences and in other case promoter seemed sufficient to drive differences of paralog expression. In *Drosophila*, a recent study of paralogous *nubbin/pdm2* genes reveals that they are controlled by the same enhancer located in the large 30 kb inter-region that is however not sufficient to drive differential expression of paralogs as promoter of *pdm2* is necessary to repress it expression is some cells [53]. Methylation profile and chromatin accessibility may also contribute to regulation of *vpg* expression. This has been demonstrated for genes encoding snake toxins [54]. Given these results, ChIP-Seq experiments could be relevant to verify the hypotheses. Those tools have not been yet developed for ants and the collection of sufficient amounts of genomic material from venom glands would be a hurdle to overcome.

## Conclusions

In this pioneering investigation on ant venom genes, we provide a high-quality assembly genome and the annotation of venom peptide genes that we think can fosters further genomic research to understand the evolutionary history of ant venom biochemistry. Based on our genomic data of *T. bicarinatum* we determined the chromosomal localization of 44 *vpg* and highlighted their organization in tandem repeat. This organization reflects the evolution of the *vpg* through local duplications from ancestor genes while the ML analysis suggests specific ancestor genes for each precursor family. Each family is dominated by the expression of one gene or one group of genes that may be considered as the most ancient *vpg*. GATA TFs BS frequency on *vpg* promoters suggest that GATA TF may be the major TF controlling *vpg* expression, and as these factors already controlled in *Drosophila* the expression of genes encoding short peptides involved in defence (HDPs), we inferred that the *vpg* ancestors may have been co-opted from genes encoding peptides that were involved in immune processes. Our study demonstrates the relevance of genomic approaches in the context of venom function to gain insight into evolutionary processes that lead to the recruitment and fixation of genes evolving through duplication processes.

## Methods

### Genomic DNA extraction

A hundred *T. bicarinatum* larvae (about 140 mg) were isolated from the same colony and divided into 20 mg samples. Genomic DNA was then extracted following the protocol of Winnepenninckx et al. with some modifications [55]. Each sample was lysed in 500 µL pre-heated Cationic detergent cetyltrimethylammonium bromide (CTAB) buffer with a pestle and treated with proteinase K (1 mg/mL) at 65 °C for 4 h. Extraction was then performed by adding an equal volume of chloroform: isoamylalcohol (24:1) to the CTBA/proteinase K solution. The aqueous phase was transferred to a new tube and the DNA was precipitated by adding one volume of isopropanol. The DNA was then washed with a 70% ethanol solution and, after air drying, dissolved in 50 µL ultrapure water. RNAse treatment was then performed at 37 °C, before repeating the extraction, precipitation and washing steps. The genomic DNA was finally redissolved in 50 µL ultrapure water, and its integrity was assessed by gel 0.8% agarose gel electrophoresis before being sent to the GeT-PlaGe core facility for sequencing.

### Long and short reads sequencing

#### Nanopore sequencing ONT 1D LSK109

Library preparation and sequencing were performed at the GeT-PlaGe core facility, INRAe Toulouse, according to the manufacturer’s instructions “1D gDNA selecting for long reads (SQK-LSK109)”. At each step, DNA was quantified using the Qubit dsDNA HS Assay Kit (Life Technologies). DNA purity was assessed using the nanodrop (Thermofisher) and size distribution and degradation were assessed using the fragment analyser (AATI) High Sensitivity DNA Fragment Analysis Kit. Purification steps were performed using AMPure XP beads (Beckman Coulter).

Flow cell was run with 7 µg of DNA previously purified and sheared to 25 kb using the megaruptor system (diagenode). Calibration was performed using the Short Read Eliminator Family: SRE size XS kit (Circulomics) to deplete short fragments. A one step DNA damage repair + END-repair + dA tail of double stranded DNA fragments was performed on 2 µg of sample. Adapters were then ligated to the library. The library was loaded onto R9.4.1 revD flow cells and sequenced on the GridION instrument (Oxford Nanopore Technologies) at 0.025pmol within 72 h.

### DNAseq Illumina HiSeq

DNAseq was performed at the GeT-PlaGe core facility, INRAe Toulouse. DNA-seq libraries were prepared according to Illumina protocols using the Illumina TruSeq Nano DNA LT Library Prep Kit. Briefly, DNA was fragmented by sonication and adapters were ligated for sequencing. Eight PCR cycles were applied to amplify the libraries. The quality of the libraries was assessed using an Advanced Analytical Fragment Analyzer and libraries were quantified by QPCR using the Kapa Library Quantification Kit. DNA-seq experiments were performed on an Illumina HiSeq using a paired-end length read of 2 × 150 bp with the Illumina HiSeq3000.

### Hi-C library

Hi-C library was constructed using the Arima-HiC kit (Arima, ref. A510008) and the Accel NGS 2S Plus DNA Library Kit (Swift Biosciences, ref.21024). Briefly, we crushed 50 whole ants in liquid nitrogen using a mortar and the resulting ground tissues were crosslinked using a 2% formaldehyde solution. After tissue lysis, we digested the crosslinked DNA according to the manufacturer’s protocol.

We repaired the digested DNA using biotinylated nucleotides and performed a ligation targeting the proximal digested ends of the DNA. We purified the proximally ligated DNA, sonicated it using a e220 focused-ultrasonicator (Covaris) and enriched the biotinylated fragments. Starting from the enriched biotinylated fragments, we constructed a NGS library using the Accel-NGS 2S Plus DNA library kit (Swift Biosciences, Ref. 21,024) according to ARIMA’s instruction. Briefly, we repaired the fragments and ligated indexed adapters to the repaired ends. After purification, a small fraction of the indexed DNA was used to determine by qPCR the number of PCR cycles required for optimal amplification. Based on this result, 6 cycles PCR amplification were performed on the remaining indexed DNA. The size distribution of the resulting libraries was monitored using a Fragment Analyzer with the High Sensitivity NGS kit (Agilent Technologies, Santa Clara, CA, USA) and the libraries were quantified by microfluorimetry (Qubit dsDNA HS kit, Thermofischer scientific). The library was denatured with NaOH, neutralized with Tris–HCl, and diluted to 1,8 pM. Clustering and sequencing were performed on a Miniseq (Illumina, San Diego, CA, USA) using the paired-end 2*150 nt protocol on a High Output flow cell. Image analyses and base calling were performed using the Miniseq Control Software and the Real-Time Analysis component (Illumina). Demultiplexing [and trimming were]/[was] performed using Illumina's conversion software (bcl2fastq 2.20). Raw data quality was assessed using FastQC (v0.11.8) from the Babraham Institute and the Illumina’s Sequencing Analysis Viewer (SAV) software. FastqScreen was used to identify potential contamination.

### Genome assembly

The quality of Illumina paired-end and Nanopore sequencing reads was assessed using fastqc (https://www.bioinformatics.babraham.ac.uk/projects/fastqc/). Nanopore reads were filtered to a minimum length of 10kb using an in-house script. Nanopore and Illumina PE reads were assembled using MaSurCa version 3.3.1 [56] using default settings. Assembly metrics were computed using the assemblathon_stats.pl script (https://github.com/KorfLab/Assemblathon). Assembly quality was checked using Benchmarking universal single-copy orthologues (BUSCO) [57] version 3.0.2 using the insecta_odb9 database and fly as species and by generating a KAT kmer spectra-cn plot [58]. The Hi-C reads (see Sect. 2.3) were then aligned to the contigs using juicer [59] with default parameters. A candidate assembly was generated using the 3D de novo assembly (3D-DNA) pipeline with the -r 0 parameter [60]. Finally, the assembly was manually reviewed using juicebox assembly tool [59].

### Structural and functional genome annotation

Specific repeats were searched for in the assembly using RepeatModeler version 2.0.1 [61] and the assembly was smoothly masked using RepeatMasker version 4.0.7 [62] with the fasta file yielded. Both software packages were run with default parameters. Maker version 3.01.02-beta-MPI was used for automatic annotation. Transcriptome annotation was performed using version 3.01.02-beta if the Maker3 genome annotation pipeline [63]. It combines annotations and evidence from three approaches: (1) similarity to ant proteins, assembled transcripts (see below), and de novo gene predictions. *Solenopsis invicta* protein sequences found at NCBI were aligned to the masked genome using exonerate version 2.2.0 [64] with the protein2genome alignment model which allows translated alignments with intron modelling. (2) RNA-seq reads from two public *Tetramorium bicarinatum* runs (SRR1106144 and SRR1106145) were aligned to the chromosomal assembly using HISAT2 version 2.2.1 [65]. The Bam files were merged using samtools merge and the wiggle signal files were generated using STAR version 2.5.1b [66] in run inputAlignmentsFromBAM mode with outWigType and outWigStrand options. Cufflinks version 2.2.1 [67] was used to assemble the transcripts, which were used as RNA-seq evidence. (3) BRAKER version 2.0.4 [68] produced de novo gene models in the form of a gff file as well as protein and transcript fasta files. The best supported transcript for each gene was selected using the annotation edit distance (AED) quality metric.

The structural annotation of the genes encoding venom peptides was performed manually. Indeed, putative mRNAs for these genes were not generated by the automatic procedure described above. The tblastn analysis using the amino acid sequences of the venom peptide precursors as input allowed us to detect the chromosomal regions containing the venom peptide genes (*vpg*). We then mapped the *Tetramorium bicarinatum* SRA transcriptomic data available in the online database (accession number: SRR1106145 and SRR1106144) using STAR-2.7.9a, to determine the intron/exon junctions of *vpg* and 5’UTR and 3’UTR positions. We used IGV_2.8.0 as genome viewer. We then created a gff file containing all the necessary information for all *vpg* and merged this file with the gff obtained after the automatic structural annotation of the genome. TATA boxes were predicted using TSS finder or manually annotated. Genome representation was performed using Tbtools V1.098774.

### Gene sequence analysis

The gene sequences were extracted by using the command gff3_to_fasta of gff3toolkit suite, the promoter sequences with extract-promoter-sequences (GitHub), and the intron sequences with the package intronIC V1.3.2 + 5 (https://github.com/glarue/intronIC). The structure of precursors encoded by the new *vpg* was predicted with Phobius (https://phobius.sbc.su.se/) and edited with Tbtools V1.098774. To compare introns, UTRs and promoters of each precursor family, sequences were aligned with ClustalW and we generated a % identity matrix from these alignments in Ugene version 43.0. Gene sequences were aligned on Ugene 43.0 with ClustalW and analysed on MEGA 11.0.13 [69] to find the best evolutionary model. The analysis was performed by maximum likelihood method and Tamura 3-parameter model (bootstrapping to 100).

### Expression profile and promoter analysis

The gff file was converted to gtf files using gffread v0.11.7 and used to accurately determine vpg and other genes levels of expression in venom glands using STAR-2.7.9a and subread-1.6.0. Promoter sequences were analysed with Jaspar (https://jaspar.genereg.net/) or with weight matrix retrieved on from the Fly factor survey database (https://pgfe.umassmed.edu/TFDBS/) by Ugene version 43.0 (Berg and von Hippel algorithm, score above 85%). The *Drosophila* matrices were validated by analysing the promoters of *T. bicarinatum* homologs of known *Drosophila* TF target genes (see supplementary M&M). To assess the possible over representation of TF BS encoded by highly expressed genes in *vpg* promoters, we compared their frequency on *vpg* promoters with their frequency on all *T. bicarinatum* gene promoters (-1000 kb above TSS). The representation of the promoter structure was performed with on Tbtools V1.098774. We also subjected some proximal promoters to an Amadeus v1.0 analysis [70] (http://acgt.cs.tau.ac.il/amadeus/) to find specific motif enrichment compared to other *T. bicarinatum* gene promoters. Since introns may also be involved in the regulation of gene expression, we also analysed their sequence to find potential BS.

### Functional annotation of the most expressed genes

The list of genes with the highest TPM was retrieved from the quantification analyses, and the corresponding ORF, was extracted from the total ORF file of *T. bicarinatum*. These ORF were subjected to blastp analysis using the *Drosophila* proteome as the database (uniprot_proteome_UP000000803) or the uniprot_sprot database.

### Supplementary Information

**Additional file 1: Figure S0.** Quality of genome assembly. A) Comparison of BUSCO metrics between contigs and chromosomes. B) Chromosomes kmer content graph: black areas correspond to kmer present in the reads and not in the chromosomes, red areas correspond to kmer present in reads and in assembled chromosomes. C) Assembly metrics. D) Hi-C map: red dots correspond to Hi-C links between two contigs, green squares correspond to contigs boundaries, blue square show chromosomes boundaries, blue histograms show Hi-C read coverage. **Figure S1.** Venom peptide gene nomenclature system used in this manuscript. The venom peptide gene name is divided into five parts describing toxin origin (red), venom gene family (blue), species source (green),  toxin peptide family (purple), and paralog (black).This nomenclature is derived from King et al. where the pharmacological descriptor was not included due to the scarcity of characterized activity and known molecular target in ant venoms. **Figure S2.** Structures and sequences of additional venom peptide precursors identified in the genome. **Figure S3.** Alignment of MYRTX_A4_-Tb21a (U21-MYRTX-Tb1a) and other precursors of A4 family found in *Tetramorium africanum*, *Manica rubida *and *Myrmica ruginodis*. Consensus sequence is above the alignment. **Figure S4.** Structure of *MYRTXA4-Tb1b *gene deduced from SRA alignment and comparison with gene structure predicted by maker. **Figure S5.** Phase-2 introns 1 of A1 *vpg *coding mature peptides with one disulfide bond and structure of new *MYRTXA1-Tb18a *transcript (mRNA2) coding MYRTXA1-Tb7b mature peptide. Detail of the Alternative splicing site in *MYRTXA1-Tb18a *exon 2. Splicing sites are boxed in red and codon in black. **Figure S6.** 5’ non canonical 5’ splicing site of *U3-Tb1a *gene. Non canonical 3’Splicing site is boxed in red. **Figure S7.** Structure of *MYRTXA3-Tb6d *gene deduced from SRA mapping. **Figure S8.** Evolutionary analysis by Maximum Likelihood method of C1/A4, B1 and B2 *vpg. *The evolutionary history was inferred by using the Maximum Likelihood method and Tamura 3-parametermodel. The tree with the highest log likelihood (-7773.71) is shown. The percentage of trees in which the associated taxa clustered together is shown next to the branches. Initial tree(s) for the heuristic search were obtained automatically by applying Neighbor-Join and BioNJ algorithms to a matrix of pairwise distances estimated using the Tamura 3 parameter model, and then selecting the topology with superior log likelihood value. A discrete Gamma distribution was used to model evolutionary rate differences among sites (5 categories  (+*G*, parameter = 3.5322)). This analysis involved 23 nucleotide sequences. All positions with less than 95% site coverage were eliminated, i.e., fewer than 5% alignment gaps, missing data, and ambiguous bases were allowed at any position (partial deletion option). There were a total of 489 positions in the final dataset. Evolutionary analyses were conducted in MEGA11. **Figure S9.** Evolutionary analysis by Maximum Likelihood method of A1, A2, A3 and B1 vpg. The evolutionary history was inferred by using the Maximum Likelihood method and Tamura 3-parameter model. The tree with the highest log likelihood (-3703.05) is shown. The percentage of trees in which the associated taxa clustered together is shown next to the branches. Initial tree(s) for the heuristic search were obtained automatically by applying Neighbor-Join and BioNJ algorithms to a matrix of pairwise distances estimated using the Tamura 3 parameter model, and then selecting the topology with superior log likelihood value. A discrete Gamma distribution was used to model evolutionary rate differences among sites (5 categories  (+*G*, parameter = 4.8238)). This analysis involved 26 nucleotide sequences. All positions with less than 95% site coverage were eliminated, i.e., fewer than 5% alignment gaps, missing data, and ambiguous bases were allowed at any position (partial deletion option). There were a total of 216 positions in the final dataset. Evolutionary analyses were conducted in MEGA11. **Figure S10.** Hits pattern on *MYRTXA2-Tb1a* and *MYRTXA2-Tb1b* genes. Numbers in the left corner indicate the hits ranges. **Figure S11.** Structure of *vpg* promoters (1000 bp upstream the TSS).

**Additional file 2: Table S0.** Correspondence of gene names to previously published peptide names. **Table S1.** Mean Id% of the different functional regions of vpg. **Table S2.** Mean Id% along the promoter of vpg families. **Table S3.** Expression levels of vpg in venom glands. **Table S4.** Gene encoding TF with TPM over 40 and evalue of annotation over 10-10. TPM in the rest of the abdomen is also indicated (TPM body) as the ratio of expression between venom gland and abdomen (Ratio). Matrix indicate if matrices are available and the site where to find them (FFS : fly factor survey database, jaspar : jaspar database). **Table S5.** Frequencies of TFBS on promoters (1000 pb among the TSS) of all T bicarinatum genes compared to specific frequencies on promoters of each vpg families. For CnC BS frequencies have been evaluated within the 500 pb among TSS. In bold : frequencies above random ones. **Table S6.** Enriched motif predicted by amadeus on A1 promoters (-400 pb among the TSS, motif length 10, all Jaspar Database/Drosophila database, Enrichment analysis).**Table S7.** Enriched motif predicted by amadeus on A2 promoters, -400 pb among the TSS, motif length 12, all Jaspar Database/Drosophila database, Enrichment analysis. **Table S8.** Frequencies of TFBS on promoters (1000 pb among the TSS) of all T bicarinatum genes compared to specific frequencies on promoters of selected TG. For CnC BS frequencies have been evaluated within the 500 pb among TSS. In bold : frequencies above random ones.

**Additional file 3. **Materials and methods (SI).

## Data Availability

The datasets generated during the current study are available on European Nucleotide Archive database with the accession number PRJEB47619 on https://www.ebi.ac.uk/ena/browser/view/PRJEB47619.

## References

[1] Schendel V, Rash LD, Jenner RA, Undheim EAB (2019). The diversity of venom: The importance of behavior and venom system morphology in understanding its ecology and evolution. Toxins.

[2] Casewell NR, Wüster W, Vonk FJ, Harrison RA, Fry BG (2013). Complex cocktails: The evolutionary novelty of venoms. Trends Ecol Evol.

[3] Tasoulis T, Pukala TL, Isbister GK (2022). Investigating Toxin Diversity and Abundance in Snake Venom Proteomes. Front Pharmacol.

[4] Walker AA (2020). The evolutionary dynamics of venom toxins made by insects and other animals. Biochem Soc Trans.

[5] Walker AA, Madio B, Jin J, Undheim EAB, Fry BG, King GF (2017). Melt with this kiss: Paralyzing and liquefying venom of the Assassin bug Pristhesancus plagipennis (Hemiptera: Reduviidae). Mol Cell Proteomics.

[6] Walker AA, Robinson SD, Paluzzi JPV, Merritt DJ, Nixon SA, Schroeder CI (2021). Production, composition, and mode of action of the painful defensive venom produced by a limacodid caterpillar, Doratifera vulnerans. Proc Natl Acad Sci USA.

[7] Walker AA, Dobson J, Jin J, Robinson SD, Herzig V, Vetter I (2018). Buzz kill: Function and proteomic composition of venom from the giant assassin fly Dolopus genitalis (Diptera: Asilidae). Toxins.

[8] Drukewitz SH, Bokelmann L, Undheim EAB, Von Reumont BM (2019). Toxins from scratch? Diverse, multimodal gene origins in the predatory robber fly Dasypogon diadema indicate a dynamic venom evolution in dipteran insects. GigaScience.

[9] Martinson EO, Kelkar YD, Chang CH, Werren JH, Mrinalini (2017). The evolution of venom by co-option of single-copy genes. Curr Biol.

[10] Koludarov I, Velasque M, Senoner T, Timm T, Greve C, Hamadou AB (2023). Prevalent bee venom genes evolved before the aculeate stinger and eusociality. BMC Biol.

[11] Touchard A, Aili SR, Fox EGP, Escoubas P, Orivel J, Nicholson GM (2016). The biochemical toxin arsenal from ant venoms. Toxins.

[12] GODFRAY HCJ. The Immature Parasitoid. In: Parasitoids. Princeton University Press; 1994. p. 225–59.

[13] Robinson SD, Mueller A, Clayton D, Starobova H, Hamilton BR, Payne RJ (2018). A comprehensive portrait of the venom of the giant red bull ant, Myrmecia gulosa, reveals a hyperdiverse hymenopteran toxin gene family. Sci Adv.

[14] von Reumont BM, Dutertre S, Koludarov I. Venom profile of the European carpenter bee Xylocopa violacea: Evolutionary and applied considerations on its toxin components. Toxicon: X. 2022;14:100117. 10.1016/j.toxcx.2022.100117 PMC8927852 35309263

[15] Perez-Riverol A, dos Santos-Pinto JRA, Lasa AM, Palma MS, Brochetto-Braga MR (2017). Wasp venomic: Unravelling the toxins arsenal of Polybia paulista venom and its potential pharmaceutical applications. J Proteomics.

[16] Jensen T, Walker AA, Nguyen SH, Jin AH, Deuis JR, Vetter I (2021). Venom chemistry underlying the painful stings of velvet ants (Hymenoptera: Mutillidae). Cell Mol Life Sci.

[17] Guido-Patiño JC, Plisson F. Profiling hymenopteran venom toxins: Protein families, structural landscape, biological activities, and pharmacological benefits. Toxicon: X. 2022;14:100119. 10.1016/j.toxcx.2022.100119 PMC8971319 35372826

[18] Touchard A, Téné N, Song PCT, Lefranc B, Leprince J, Treilhou M (2018). Deciphering the molecular diversity of an ant venom peptidome through a venomics approach. J Proteome Res.

[19] Barassé V, Touchard A, Téné N, C K, Paquet F, Tysklind N, et al. Venomics survey of six myrmicine ants provides insights into the molecular and structural diversity of their peptide toxins. Insect Biochem Mol Biol. 2022; 151:103876 10.1016/j.ibmb.2022.103876 36410579

[20] Touchard A, Aili SR, Téné N, Barassé V, Klopp C, Dejean A (2020). Venom peptide repertoire of the European myrmicine ant Manica rubida: Identification of insecticidal toxins. J Proteome Res.

[21] Bonnafé E, Téné N, Berger F, Rifflet A, Guilhaudis L, Ségalas-Milazzo I (2016). Biochemical and biophysical combined study of bicarinalin, an ant venom antimicrobial peptide. Peptides.

[22] Duraisamy K, Singh K, Kumar M, Lefranc B, Bonnafé E, Treilhou M (2022). P17 induces chemotaxis and differentiation of monocytes via MRGPRX2-mediated mast cell–line activation. J Allergy Clin Immunol.

[23] Hadzić T, Park D, Abruzzi KC, Yang L, Trigg JS, Rohs R (2015). Genome-wide features of neuroendocrine regulation in Drosophila by the basic helix-loop-helix transcription factor DIMMED. Nucleic Acids Res.

[24] Johnson  DM, Wells MB, Fox R, Lee JS, Loganathan R, Levings D (2020). CrebA increases secretory capacity through direct transcriptional regulation of the secretory machinery, a subset of secretory cargo, and other key regulators. Traffic.

[25] Petersen UM, Kadalayil L, Rehorn KP, Hoshizaki DK, Reuter R, Engström Y (1999). Serpent regulates Drosophila immunity genes in the larval fat body through an essential GATA motif. EMBO J.

[26] Senger K, Harris K, Levine M (2006). GATA factors participate in tissue-specific immune responses in Drosophila larvae. Proc Natl Acad Sci USA.

[27] Rus F, Flatt T, Tong M, Aggarwal K, Okuda K, Kleino A (2013). Ecdysone triggered PGRP-LC expression controls Drosophila innate immunity. EMBO J.

[28] Senger K, Armstrong GW, Rowell WJ, Kwan JM, Markstein M, Levine M (2004). Immunity regulatory DNAs share common organizational features in Drosophila. Mol Cell.

[29] Myllymäki H, Rämet M (2014). JAK/STAT Pathway in Drosophila immunity. Scand J Immunol.

[30] Minakhina S, Tan W, Steward R (2011). JAK/STAT and the GATA factor Pannier control hemocyte maturation and differentiation in Drosophila. Dev Biol.

[31] Loboda A, Damulewicz M, Pyza E, Jozkowicz A, Dulak J (2016). Role of Nrf2/HO-1 system in development, oxidative stress response and diseases: an evolutionarily conserved mechanism. Cell Mol Life Sci.

[32] Suryamohan K, Krishnankutty SP, Guillory J, Jevit M, Schröder MS, Wu M (2020). The Indian cobra reference genome and transcriptome enables comprehensive identification of venom toxins. Nat Genet.

[33] Hargreaves AD, Swain MT, Hegarty MJ, Logan DW, Mulley JF (2014). Restriction and recruitment-gene duplication and the origin and evolution of snake venom toxins. Genome Biol Evol.

[34] Ashwood LM, Elnahriry KA, Stewart ZK, Shafee T, Naseem MU, Szanto T (2023). Genomic, functional and structural analyses elucidate evolutionary innovation within the sea anemone 8 toxin family. BMC Biol.

[35] Gendreau KL, Haney RA, Schwager EE, Wierschin T, Stanke M, Richards S (2017). House spider genome uncovers evolutionary shifts in the diversity and expression of black widow venom proteins associated with extreme toxicity. BMC Genomics.

[36] Pardos-Blas JR, Irisarri I, Abalde S, Afonso CML, Tenorio MJ, Zardoya R (2021). The genome of the venomous snail Lautoconus ventricosus sheds light on the origin of conotoxin diversity. GigaScience.

[37] Robinson ASD, Deuis JR, Touchard A, Keramidas A, Mueller A, Schroeder C (2023). Ant venoms contain vertebrate-specific pain-causing sodium channel toxins. Nat Comm.

[38] Shew CJ, Carmona-Mora P, Soto DC, Mastoras M, Roberts E, Rosas J (2021). Diverse molecular mechanisms contribute to differential expression of Human duplicated genes. Mol Biol Evol.

[39] Lan X, Pritchard JK (2016). Coregulation of tandem duplicate genes slows evolution of subfunctionalization in mammals. Science.

[40] Kocabas A, Duarte T, Kumar S, Hynes MA (2015). Widespread differential expression of coding region and 3’ UTR sequences in neurons and other tissues. Neuron.

[41] Ji S, Yang Z, Gozali L, Kenney T, Kocabas A, Park CJ (2021). Distinct expression of select and transcriptome-wide isolated 3’UTRs suggests critical roles in development and transition states. PLoS ONE.

[42] Ascoët S, Touchard A, Téné N, Lefranc B, Leprince J, Paquet F (2023). The mechanism underlying toxicity of a venom peptide against insects reveals how ants are master at disrupting membranes. iScience.

[43] Lee KS, Kim BY, Yoon HJ, Choi YS, Jin BR (2016). Secapin, a bee venom peptide, exhibits anti-fibrinolytic, anti-elastolytic, and anti-microbial activities. Dev Comp Immunol.

[44] Baracchi D, Francese S, Turillazzi S (2011). Beyond the antipredatory defence: Honey bee venom function as a component of social immunity. Toxicon.

[45] Baracchi D, Mazza G, Turillazzi S (2012). From individual to collective immunity: The role of the venom as antimicrobial agent in the Stenogastrinae wasp societies. J Insect Physiol.

[46] Lynch M, O’Hely M, Walsh B, Force A (2001). The probability of preservation of a newly arisen gene duplicate. Genetics.

[47] Jackson TNW, Koludarov I (2020). How the toxin got its toxicity. Front Pharmacol.

[48] Casewell NR, Wagstaff SC, Harrison RA, Renjifo C, Wuster W (2011). Domain loss facilitates accelerated evolution and neofunctionalization of duplicate snake venom metalloproteinase toxin Genes. Mol BiolEvol.

[49] Lynch VJ (2007). Inventing an arsenal: adaptive evolution and neofunctionalization of snake venom phospholipase A2 genes. BMC Evol Biol.

[50] Gopalan SS, Perry BW, Schield DR, Smith CF, Mackessy SP, Castoe TA (2022). Origins, genomic structure and copy number variation of snake venom myotoxins. Toxicon.

[51] Ye X, Yang Y, Zhao C, Xiao S, Sun YH, He C (2022). Genomic signatures associated with maintenance of genome stability and venom turnover in two parasitoid wasps. Nat Commun.

[52] Peters RS, Krogmann L, Mayer C, Donath A, Gunkel S, Meusemann K (2017). Evolutionary History of the Hymenoptera. Curr Biol.

[53] Loker R, Mann RS (2022). Divergent expression of paralogous genes by modification of shared enhancer activity through a promoter-proximal silencer. Curr Biol.

[54] Margres MJ, Rautsaw RM, Strickland JL, Mason AJ, Schramer TD, Hofmann EP (2021). The Tiger Rattlesnake genome reveals a complex genotype underlying a simple venom phenotype. Proc Natl Acad Sci USA.

[55] Winnepenninckx B, Backeljau T, De Wachter R (1993). Extraction of high molecular weight DNA from molluscs. Trends Genet.

[56] Zimin AV, Puiu D, Luo M-C, Zhu T, Koren S, Marçais G (2017). Hybrid assembly of the large and highly repetitive genome of Aegilops tauschii, a progenitor of bread wheat, with the MaSuRCA mega-reads algorithm. Genome Res.

[57] Simão FA, Waterhouse RM, Ioannidis P, Kriventseva EV, Zdobnov EM (2015). BUSCO: assessing genome assembly and annotation completeness with single-copy orthologs. Bioinformatics.

[58] Mapleson D, Garcia Accinelli G, Kettleborough G, Wright J, Clavijo BJ (2017). KAT: a K-mer analysis toolkit to quality control NGS datasets and genome assemblies. Bioinformatics.

[59] Durand NC, Shamim MS, Machol I, Rao SSP, Huntley MH, Lander ES (2016). Juicer provides a one-click system for analyzing loop-resolution Hi-C experiments. Cell Syst.

[60] Dudchenko O, Batra SS, Omer AD, Nyquist SK, Hoeger M, Durand NC (2017). De novo assembly of the Aedes aegypti genome using Hi-C yields chromosome-length scaffolds. Science.

[61] Flynn JM, Hubley R, Goubert C, Rosen J, Clark AG, Feschotte C (2020). RepeatModeler2 for automated genomic discovery of transposable element families. Proc Natl Acad Sci USA.

[62] Tarailo‐Graovac M, Chen N. Using RepeatMasker to identify repetitive elements in genomic Sequences. CP in Bioinformatics. 2009;Mar Chapter 4:4.10.1–4.10.14. 10.1002/0471250953.bi0410s25 19274634

[63] Cantarel BL, Korf I, Robb SMC, Parra G, Ross E, Moore B (2008). MAKER: An easy-to-use annotation pipeline designed for emerging model organism genomes. Genome Res.

[64] Slater G, Birney E (2005). Automated generation of heuristics for biological sequence comparison. BMC Bioinformatics.

[65] Kim D, Paggi JM, Park C, Bennett C, Salzberg SL (2019). Graph-based genome alignment and genotyping with HISAT2 and HISAT-genotype. Nat Biotechnol.

[66] Dobin A, Davis CA, Schlesinger F, Drenkow J, Zaleski C, Jha S (2013). STAR: ultrafast universal RNA-seq aligner. Bioinformatics.

[67] Trapnell C, Williams BA, Pertea G, Mortazavi A, Kwan G, Van Baren MJ (2010). Transcript assembly and quantification by RNA-Seq reveals unannotated transcripts and isoform switching during cell differentiation. Nat Biotechnol.

[68] Hoff KJ, Lomsadze A, Borodovsky M, Stanke M (2019). Whole-genome annotation with BRAKER. Methods Mol Biol.

[69] Tamura K, Stecher G, Kumar S (2021). MEGA11: Molecular evolutionary genetics analysis version 11. Mol Biol Evol.

[70] Linhart C, Halperin Y, Shamir R (2008). Transcription factor and microRNA motif discovery: The Amadeus platform and a compendium of metazoan target sets. Genome Res.

