# Supplementary material for: The genome of the ant Tetramorium bicarinatum reveals a tandem organization of venom peptides genes allowing the prediction of their regulatory and evolutionary profiles
Source: BMC Genomics. 2024 Jan 20;25:84. doi: 10.1186/s12864-024-10012-y (PMC10800049; doi:10.1186/s12864-024-10012-y)
Supplement: Supplementary file 1 — Additional file 1: Figure S0. Quality of genome assembly. A) Comparison of BUSCO metrics between contigs and chromosomes. B) Chromosomes kmer content graph: black areas correspond to kmer present in the reads and not in the chromosomes, red areas correspond to kmer present in reads and in assembled chromosomes. C) Assembly metrics. D) Hi-C map: red dots correspond to Hi-C links between two contigs, green squares correspond to contigs boundaries, blue square show chromosomes boundaries, blue histograms show Hi-C read coverage. Figure S1. Venom peptide gene nomenclature system used in this manuscript. The venom peptide gene name is divided into five parts describing toxin origin (red), venom gene family (blue), species source (green), toxin peptide family (purple), and paralog (black).This nomenclature is derived from King et al. where the pharmacological descriptor was not included due to the scarcity of characterized activity and known molecular target in ant venoms. Figure S2. Structures and sequences of additional venom peptide precursors identified in the genome. Figure S3. Alignment of MYRTXA4-Tb21a (U21-MYRTX-Tb1a) and other precursors of A4 family found in Tetramorium africanum, Manica rubida and Myrmica ruginodis. Consensus sequence is above the alignment. Figure S4. Structure of MYRTXA4-Tb1b gene deduced from SRA alignment and comparison with gene structure predicted by maker. Figure S5. Phase-2 introns 1 of A1 vpg coding mature peptides with one disulfide bond and structure of new MYRTXA1-Tb18a transcript (mRNA2) coding MYRTXA1-Tb7b mature peptide. Detail of the Alternative splicing site in MYRTXA1-Tb18a exon 2. Splicing sites are boxed in red and codon in black. Figure S6. 5’ non canonical 5’ splicing site of U3-Tb1a gene. Non canonical 3’Splicing site is boxed in red. Figure S7. Structure of MYRTXA3-Tb6d gene deduced from SRA mapping. Figure S8. Evolutionary analysis by Maximum Likelihood method of C1/A4, B1 and B2 vpg. The evolutionary history was inferr [file 12864_2024_10012_MOESM1_ESM.pdf]

Supplementary Information_Figures

A)

	contigs	scaffolds
**Number**	728	288
**Total length**	276283309	257967262
**N50**	3408643	22406985
**L50**	24	5

B)

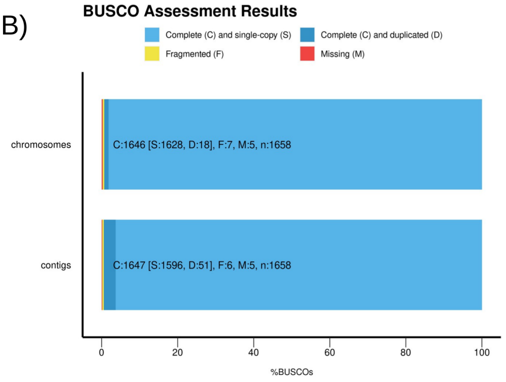

C)

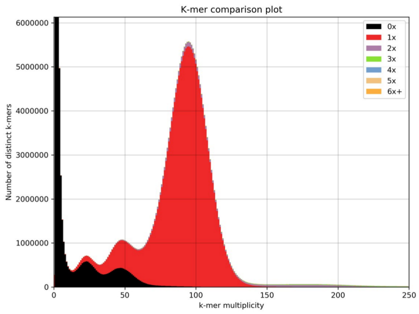

D)

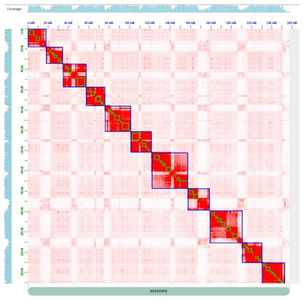

Figure S0. Quality of genome assembly. A) Comparison of BUSCO metrics between contigs and chromosomes. B) Chromosomes kmer content graph: black areas correspond to kmer present in the reads and not in the chromosomes, red areas correspond to kmer present in reads and in assembled chromosomes. C) Assembly metrics. D) Hi-C map: red dots correspond to Hi-C links between two contigs, green squares correspond to contigs boundaries, blue square show chromosomes boundaries, blue histograms show Hi-C read coverage.

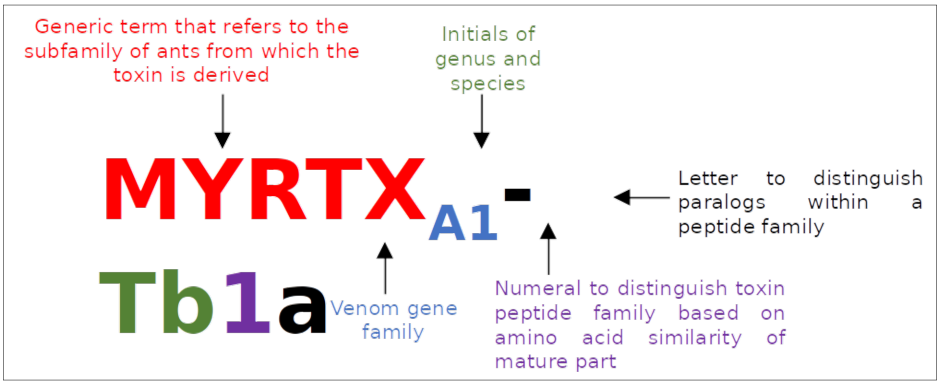

Figure S1. **Venom peptide gene nomenclature system used in this manuscript**. The venom peptide gene name is divided into five parts describing toxin origin (red), venom gene family (blue), species source (green), toxin peptide family (purple), and paralog (black).This nomenclature is derived from King et al. where the pharmacological descriptor was not included due to the scarcity of characterized activity and known molecular target in ant venoms.

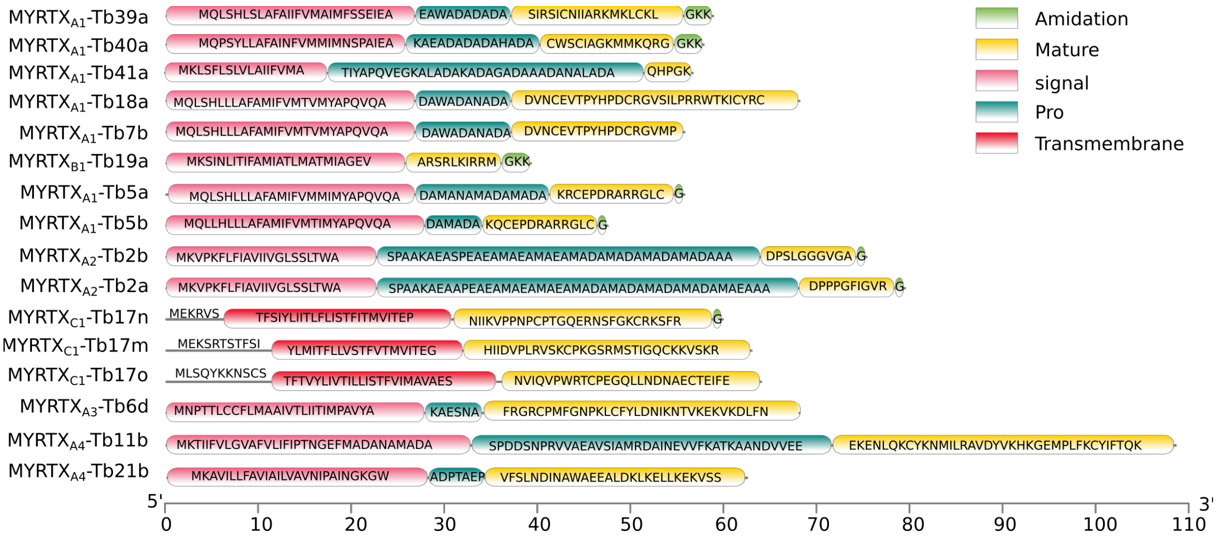

Figure S2. Structures and sequences of additional venom peptide precursors identified in the genome.

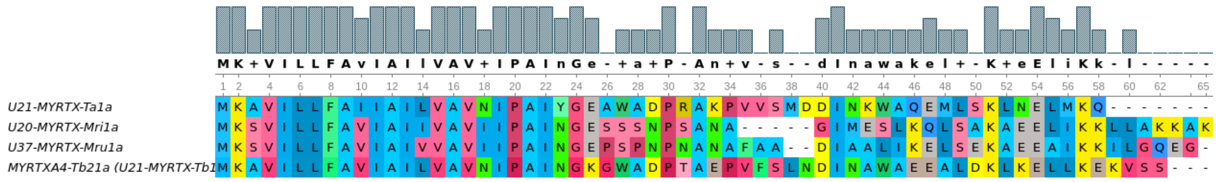

Figure S3 : Alignment of MYRTX$_{A4}$-Tb21a (U$_{21}$-MYRTX-Tb1a) and other precursors of A4 family found in *Tetramorium africanum*, *Manica rubida* and *Myrmica ruginodis*. Consensus sequence is above the alignment.

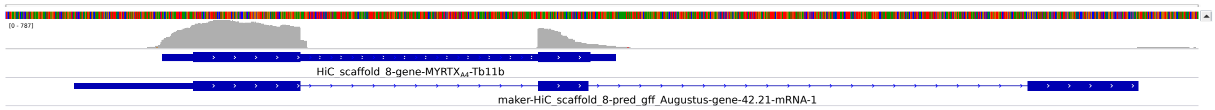

Figure S4. Structure of *MYRTX$_{A4}$-Tb1b* gene deduced from SRA alignment and comparison with gene structure predicted by maker.

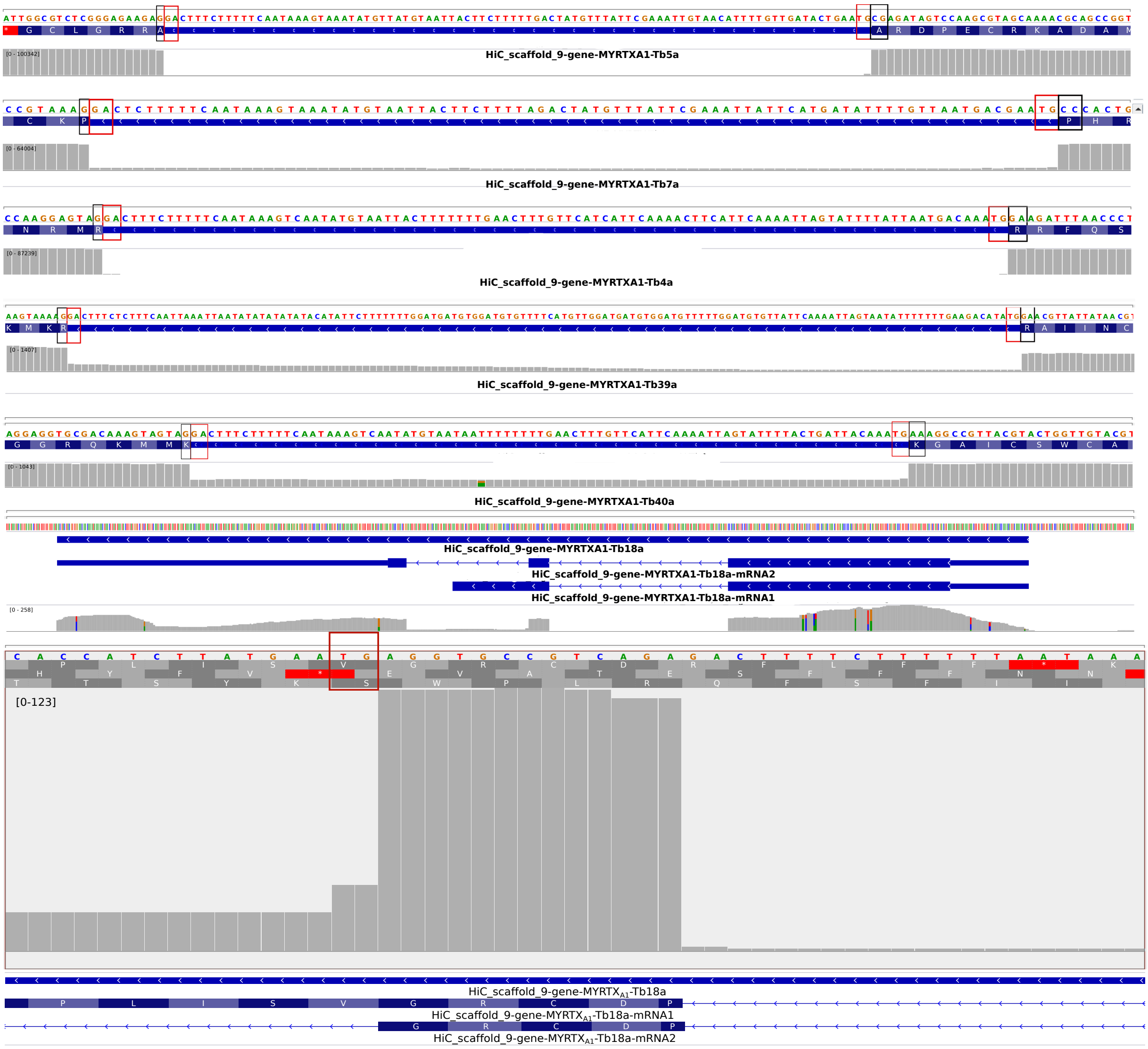

Figure S5 : Phase-2 introns 1 of A1 *vpg* coding mature peptides with one disulfide bond and structure of new *MYRTX$_{A1}$-Tb18a* transcript (mRNA2) coding MYRTX$_{A1}$-Tb7b mature peptide. Detail of the Alternative splicing site in *MYRTX$_{A1}$-Tb18a* exon 2. Splicing sites are boxed in red and codon in black.

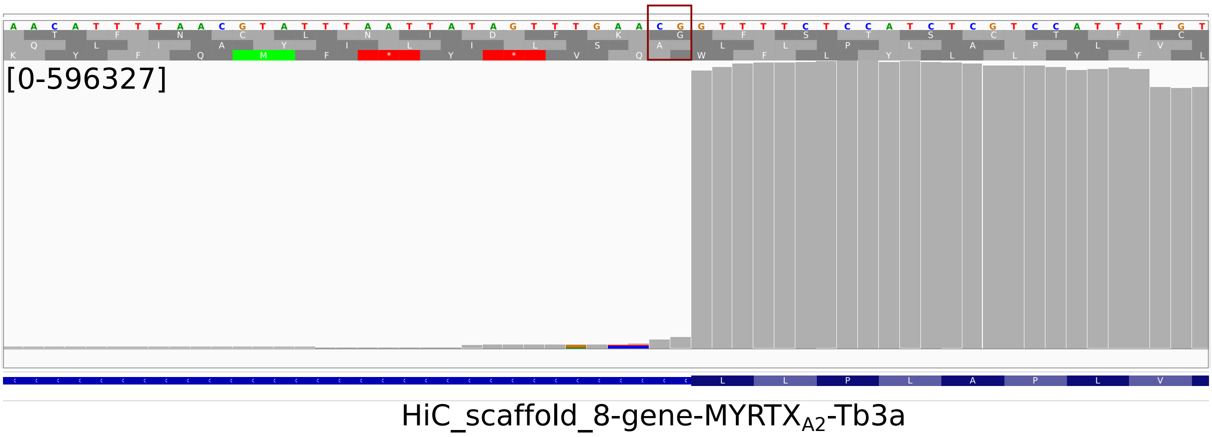

Figure S6 : 5’ non canonical 5’ splicing site of *U$_3$-Tb1a* gene. Non canonical 3’Splicing site is boxed in red

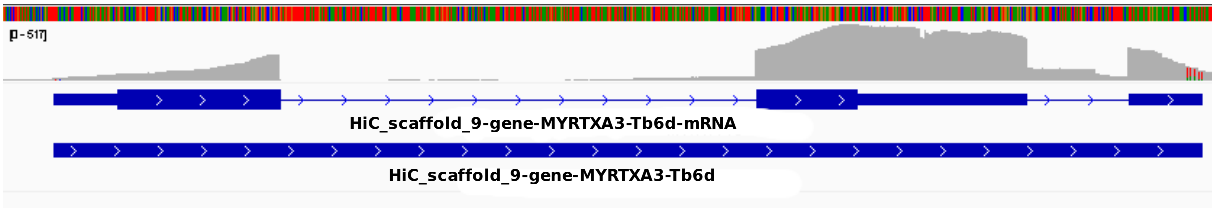

FigureS7 : structure of *MYRTX*$_{A3}$*-Tb6d* gene deduced from SRA mapping.

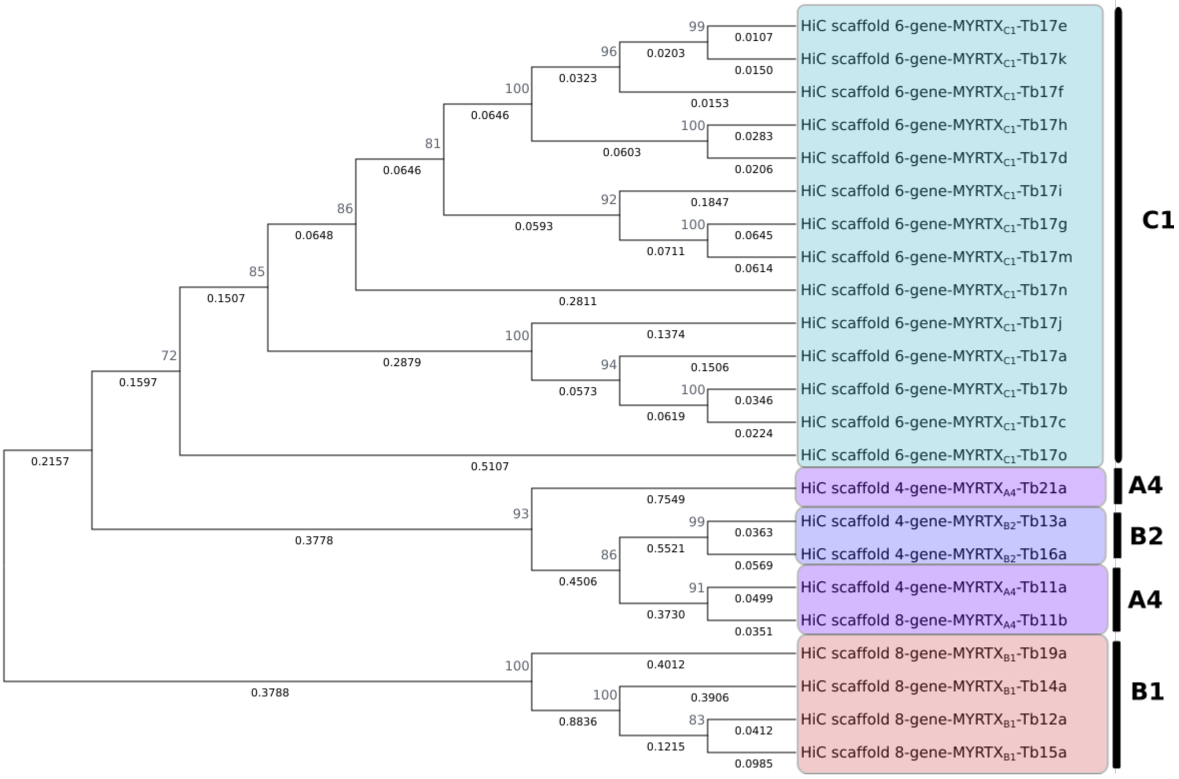

Figure S8 : Evolutionary analysis by Maximum Likelihood method of C1/A4, B1 and B2 *vpg*

The evolutionary history was inferred by using the Maximum Likelihood method and Tamura 3-parameter model. The tree with the highest log likelihood (-7773.71) is shown. The percentage of trees in which the associated taxa clustered together is shown next to the branches. Initial tree(s) for the heuristic search were obtained automatically by applying Neighbor-Join and BioNJ algorithms to a matrix of pairwise distances estimated using the Tamura 3 parameter model, and then selecting the topology with superior log likelihood value. A discrete Gamma distribution was used to model evolutionary rate differences among sites (5 categories (+*G*, parameter = 3.5322)). This analysis involved 23 nucleotide sequences. All positions with less than 95% site coverage were eliminated, i.e., fewer than 5% alignment gaps, missing data, and ambiguous bases were allowed at any position (partial deletion option). There were a total of 489 positions in the final dataset. Evolutionary analyses were conducted in MEGA11

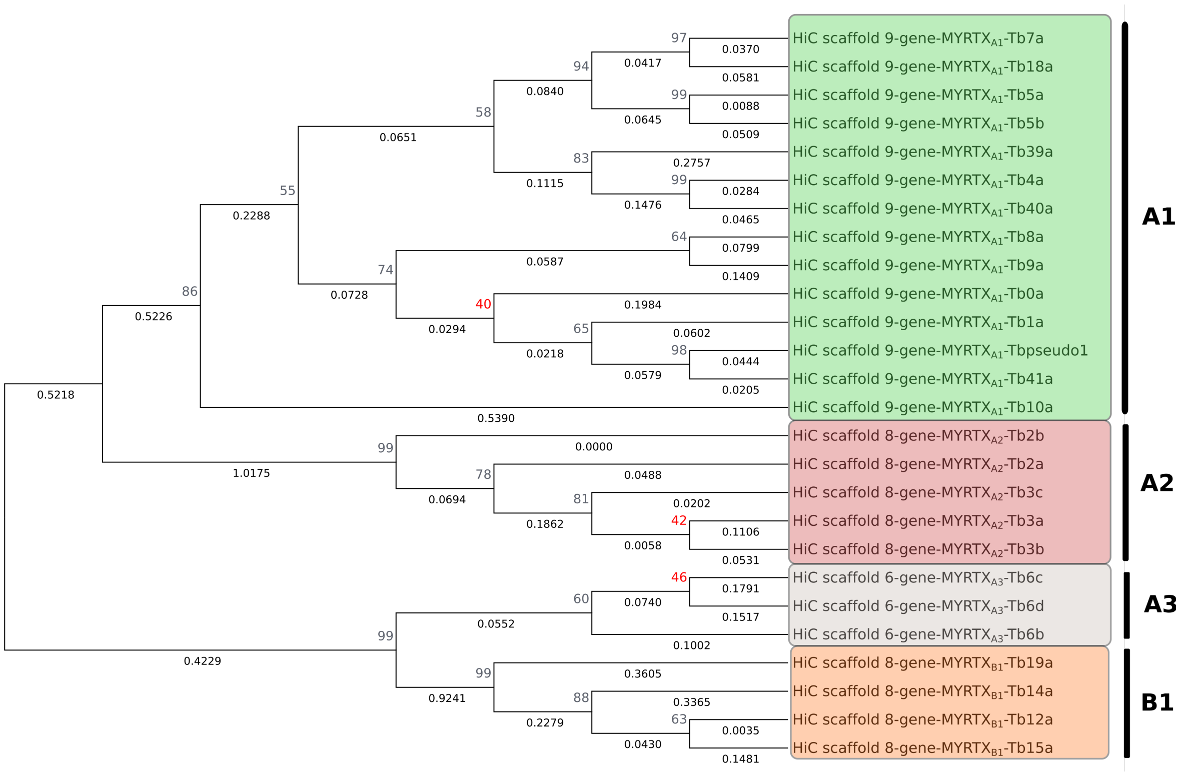

Figure S9 : Evolutionary analysis by Maximum Likelihood method of A1, A2, A3 and B1 vpg

The evolutionary history was inferred by using the Maximum Likelihood method and Tamura 3-parameter model. The tree with the highest log likelihood (-3703.05) is shown. The percentage of trees in which the associated taxa clustered together is shown next to the branches. Initial tree(s) for the heuristic search were obtained automatically by applying Neighbor-Join and BioNJ algorithms to a matrix of pairwise distances estimated using the Tamura 3 parameter model, and then selecting the topology with superior log likelihood value. A discrete Gamma distribution was used to model evolutionary rate differences among sites (5 categories (+*G*, parameter = 4.8238)). This analysis involved 26 nucleotide sequences. All positions with less than 95% site coverage were eliminated, i.e., fewer than 5% alignment gaps, missing data, and ambiguous bases were allowed at any position (partial deletion option). There were a total of 216 positions in the final dataset. Evolutionary analyses were conducted in MEGA11

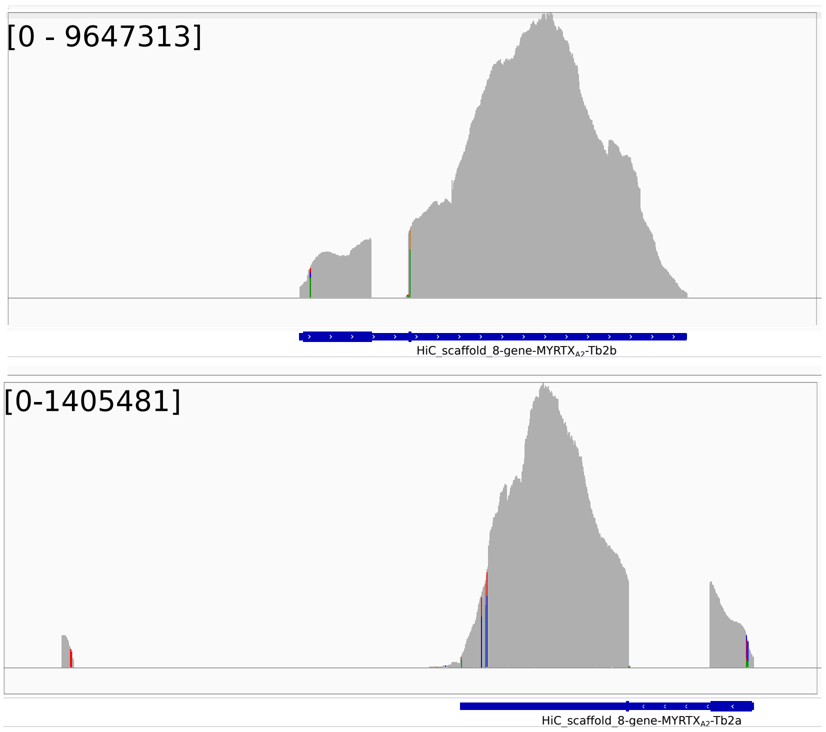

Figure S10: Hits pattern on *MYRTX*$_{A2}$*-Tb1a* and *MYRTX*$_{A2}$*-Tb1b* genes. Numbers in the left corner indicate the hits ranges.

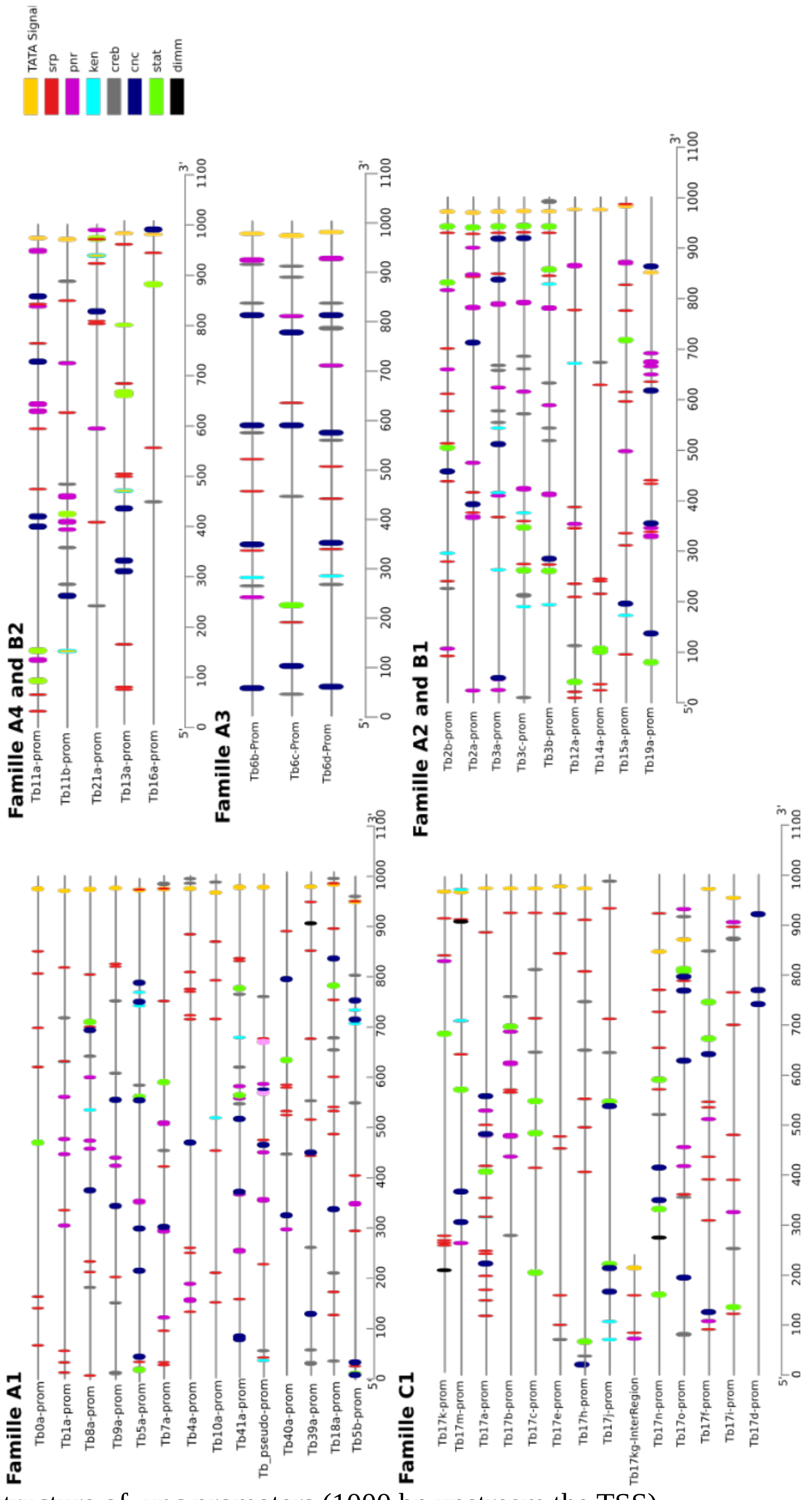

Figure S11: structure of *vpg* promoters (1000 bp upstream the TSS)
