# Supplementary material for: The genome of the ant Tetramorium bicarinatum reveals a tandem organization of venom peptides genes allowing the prediction of their regulatory and evolutionary profiles
Source: BMC Genomics. 2024 Jan 20;25:84. doi: 10.1186/s12864-024-10012-y (PMC10800049; doi:10.1186/s12864-024-10012-y)
Supplement: Supplementary file 3 — Additional file 3. Materials and methods (SI). [file 12864_2024_10012_MOESM3_ESM.pdf]

Materials and methods (SI)

TF Matrices validation

To validate drosophila matrices we analysed promoters of homologues of known target genes (TG) of selected TF (Additional_file_2, Table S8). To validate the *Drosophila* DimmED matrix we analysed the promoter and first intron sequences (DimmeD BS often localized in the first introns of TG) of *T. bicarinatum phm* homologue, a well know direct target of DimmED in *Drosophila melanogaster* [1]. Six DimmED BS were found in the first intron in reverse/forward positions and no BS in the promoter (1000 bp upstream the TSS). These results indicated that *Drosophila* matrix was relevant to predict DimmED sites in *T. bicarinatum* genes. CrebA is a leucine zipper transcription factor. CrebA matrix was designed according to [2] and validated by using promoter sequences of target SPCGs (*secretory pathway component encoding genes*). Among the 34 CrebA SPCGs listed in [2] and Tudor-SN [3], 16 have been functionally annotated and were highly expressed in venom glands. For most of them, at least one crebA BS was found near the TSS (around 100 bp upstream or downstream) and several BS along the promoter with an average number of BS (5,2) above of the random frequency (2,2).

To validate Srp and Pnr. we searched for their target gene homologues in *T. bicarinatum* genome. We did not find HDPs homologue genes in *T. bicarinatum* genome except for a defensin-like gene Tb*def1*. Two additional genes that potentially correspond to *pgrp-sb1 and one to prgrp-LC*, another target gene of *Drosophila melanogaster* Srp, were detected. The analysis of *def1* and the 3 putative *pgrp* genes promoters revealed one potential Srp BS seven Pnr BS for *def1* promoter. Six, eight and nine Srp sites and five and seven/none Pnr sites were predicted on each of the two putative *pgrp-sb1/pgrp-LC* genes promoters. The numbers of GATA BS are then clearly above their random frequency on a *T. bicarinatum* total promoters. In Drosophila, Stat92E promote the expression of *tep1* (promoting phagocytosis) and *tot* (turandot) A, C and M (stress tolerance secreted peptides) genes in response to cytokine (unpaired) that activate the receptor domeless [4]. We found one potential *tep* gene in T bicarinatum genome but no *tot* genes. The analyse of *tep* promoter revealed 5 Stat92E potential binding site which validates Stat92E matrix.

Cap'n'collar (Cnc) protein control target genes involved in the protection against oxidative stress. Seven Cnc TG were highly expressed in venom gland, three *gst* genes and four *cyp450* genes. Promoters of SOD and catalase genes which are highly expressed but do not target Cnc genes, were used as negative control [5]. The Cnc TF promoters contain 17 Cnc BS with 16 within the 500 bp before the TSS as described by [5]. The frequency on 1000 bp was not above the random frequency (1,1) on all of *T. bicarinatum* promoters. However, by considering the 500 bp among the TSS of all *T bicarinatum* promoter, Cnc BS random frequency was 2,3 on the 500 bp Cnc TG promoters. We thus decided to take in account in the analysis the position of Cnc BS on vpg promoters.

Reference (SI)

1. Park D, Shafer OT, Shepherd SP, Suh H, Trigg JS, Taghert PH. The Drosophila Basic Helix-Loop-Helix Protein DIMMED Directly Activates PHM , a Gene Encoding a Neuropeptide-Amidating Enzyme. Molecular and Cellular Biology. 2008;28:410–21.

2. Abrams EW, Andrew DJ. CrebA regulates secretory activity in the Drosophila salivary gland and epidermis. Development. 2005;132:2743–58.

3. M. Johnson D, Wells MB, Fox R, Lee JS, Loganathan R, Levings D, et al. CrebA increases secretory capacity through direct transcriptional regulation of the secretory machinery, a subset of secretory cargo, and other key regulators. Traffic. 2020;21:560–77.

4. Myllymäki H, Rämet M. JAK/STAT Pathway in Drosophila Immunity. Scandinavian Journal of Immunology. 2014;79:377–85.

5. Loboda A, Damulewicz M, Pyza E, Jozkowicz A, Dulak J. Role of Nrf2/HO-1 system in development, oxidative stress response and diseases: an evolutionarily conserved mechanism. Cellular and Molecular Life Sciences. 2016;73:3221–47.
